# Immunosuppressive treatment for idiopathic membranous nephropathy: An updated network meta-analysis

**DOI:** 10.1515/biol-2022-0527

**Published:** 2023-01-10

**Authors:** Neng Bao, Mingjia Gu, Xiang Yu, Jin Wang, Leiping Gao, Zhiwei Miao, Wei Kong

**Affiliations:** Department of Nephrology, Nanjing Hospital of Chinese Medicine Affiliated to Nanjing University of Chinese Medicine, 157 Daming Road, Nanjing City, Jiangsu, 210000, PR China; Department of Nephrology, Changshu Hospital Affiliated to Nanjing University of Chinese Medicine, 6 Huanghe Road, Changshu City, Jiangsu, 215500, PR China; Department of Nephrology, Nanjing Hospital of Chinese Medicine Affiliated to Nanjing University of Chinese Medicine, Nanjing City, Jiangsu, 210000, PR China; Department of Gastroenterology, Affiliated Hospital of Jiangnan University, 1000 Hefeng Road, Binhu District of Wuxi, Jiangsu, 214000, PR China; Department of Gastroenterology, Zhangjiagang TCM Hospital Affiliated to Nanjing University of Chinese Medicine, 77 Changan South Road, Zhangjiagang, 215600, PR China

**Keywords:** idiopathic membranous nephropathy, immunosuppressive treatment, network meta-analysis, GRADE

## Abstract

This network meta-analysis (NMA) aims to investigate the efficacy and safety of different pharmacological treatments for idiopathic membranous nephropathy (IMN). Thirty-four relevant studies were extracted from PubMed, Embase, Cochrane database, and MEDLINE. Treatment with tacrolimus (TAC), cyclophosphamide (CTX), mycophenolate mofetil, chlorambucil (CHL), cyclosporin A (CSA), steroids, rituximab (RTX), and conservative therapy were compared. Outcomes were measured using remission rate and incidence of side effects. Summary estimates were expressed as the odds ratio (OR) and 95% confidence intervals (CIs). The quality of findings was assessed using the Grading of Recommendations, Assessment, Development, and Evaluation approach. In the direct meta-analysis for comparison of complete remission (CR) rate, the curative effect of RTX is inferior to CTX (OR 0.37; CI 0.18, 0.75). In the NMA of CR rate, the results showed that the curative effects of CTX, CHL, and TAC were significantly higher than those of the control group. The efficacy of RTX is not inferior to the CTX (OR 0.81; CI 0.32, 2.01), and the level of evidence was moderate; CSA was not as effective as RTX, and the difference was statistically significant with moderate evidence (OR 2.98, CI 1.00, 8.91). In summary, we recommend CTX and RTX as the first-line drug for IMN treatment.

## 1 Introduction

In recent years, the incidence rate of idiopathic membranous nephropathy (IMN) was shown to be increasing [1]. IMN is the most prevalent cause of nephrotic syndrome in adults [2]. Although one-third of patients can recover spontaneously [3], nearly 30–40% of patients with nephrotic syndrome do not recover and succumb to end-stage renal disease [4,5]. The pathogenesis of IMN is thought to be caused by the deposition of immune complexes such as IgG and complement protein C3 in the glomerular basement membrane, resulting in the thickening of the glomerular capillary wall and proteinuria [6]. Therefore, immunosuppressive therapy is the mainstay in the treatment of IMN.

The guidelines issued by the Kidney Disease Improving Global Outcomes (KDIGO) in 2021 have been updated in the treatment of membranous nephropathy [7]. Rituximab (RTX) or calcineurin inhibitor (CNI) is recommended as the initial treatment for moderate-risk patients with normal estimated glomerular filtration rate (eGFR) and large proteinuria (greater than 3.5 g/day). For high-risk patients mostly characterized by decreased eGFR, cyclophosphamide (CTX) is recommended as the first-line drug. Compared with the previous version [8], the focus of this guideline update mainly concerns the use of RTX. RTX is a chimeric monoclonal IgG1 antibody, which reduces proteinuria by binding CD20 to consume B cells. In recent years, high-quality randomized controlled trials (RCTs) have shown that RTX is effective in the treatment of IMN, and the incidence of side effects is relatively low [9,10]. Therefore, it has become defined as another effective intervention for the therapy of IMN.

As an emerging intervention parallel to CTX and CNIs recommended by KDIGO guidelines, RTX has attracted extensive attention in academic circles in recent years. However, recently published articles show that it has no advantage in the efficacy and safety of IMN compared with CTX [11,12]. With the advent of RTX, it was necessary to re-evaluate the mainstays of treatment for IMN. Due to the lack of head-to-head RCTs of RTX, CTX, and CNIs, it is difficult for clinicians to comprehensively compare their advantages and disadvantages. A network meta-analysis (NMA) can evaluate multiple interventions simultaneously by calculating the combined effect between various measures. These are mostly based on the Bayesian or frequency methods to calculate and analyze the direct effect quantity (direct comparison of the two treatment interventions) and indirect effect quantity (comparison of the two treatment interventions with one as the reference), respectively. These results are then combined to generate a mixed effect quantity that quantifies the advantages and disadvantages of each treatment.

Therefore, we conducted an NMA to compare the efficacy and safety of common treatments for IMN such as RTX, CTX, and CNIs, and assessed the obtained effect amount according to the Grading of Recommendations Assessment Development, and Evaluation (GRADE) criteria [13,14,15,16,17,18] to provide the corresponding level of evidence for the final results.

## 2 Methods

The study protocol was registered in the International Prospective Register of Systematic Reviews (CRD42019131825) and was consistent with the statements of Preferred Reporting Items for Systematic Reviews and Meta-Analyses.

### 2.1 Inclusion criteria

The study in question must be an RCT, and the observation time shall not be less than 6 months. In addition, the following points must be met:

1) The patients must be adults (over 18 years old) diagnosed with IMN by renal biopsy.
2) Interventions in the treatment group must be either/or CTX, chlorambucil (CHL), mycophenolate mofetil (MMF), tacrolimus (TAC), cyclosporin A (CSA), RTX, or steroids (STE), of which the first five regimens can be used in combination with steroids or alone.
3) Measures in the control group included placebo/no treatment, conservative treatment (ACEI/ARB or other supportive treatment), steroids, CTX, CHL, or CSA. The latter three therapies can be used in combination with steroids or alone.
4) The results must include the rate of remission (including complete or partial remission (PR)) or the incidence of side effects, or both.

### 2.2 Exclusion criteria

The following criteria were excluded:

1) Non-randomized trials or observational studies;
2) Studies of secondary membranous nephropathy (such as hepatitis B related nephropathy);
3) Studies with patients younger than 18 years;
4) Studies with observation times less than 6 months;
5) Alternative treatments such as ACTH and leflunomide;
6) Studies using herbal or traditional Chinese medicines;
7) Studies using Azathioprine or mizoribine due to their poor efficacy [19].

### 2.3 Retrieval strategy

Two researchers (NB and XY) conducted an independent literature search for articles or abstracts. The retrieval databases include PubMed, Embase, Cochrane database, and MEDLINE. The retrieved timeline is set as August 15, 2021 as a reference. Duplicate entries were resolved through negotiation.

### 2.4 Baseline characteristics and quality assessment

Two researchers (M-JG and XY) extracted the detailed information such as study design, sample size, drug dosing, and specific characteristics of patients in the study. The results were extracted at the end of each study. When multiple points in time were reported, the last point in time was used. We used the Cochrane bias risk assessment tool to assess the quality of single literature. According to the Cochrane risk deviation assessment, the remission rate and the incidence of side effects were objective indicators. Therefore, although most of the included studies were not double-blind, it can be assumed that the above sources of bias are low. Any doubt shall be resolved through direct consultation with the expert group.

### 2.5 Observation index

Efficacy indicators include complete remission (CR) and total remission (TR). CR refers to urinary protein ≤0.3 g/24 h and stable renal function. PR refers to urinary protein >0.3 g/24 h, but <3.5 g/24 h and <50% baseline value. TR is the sum of CR and PR.

Safety indicators include hepatic and renal injury, infection, bone marrow suppression, gastrointestinal symptoms, nervous system symptoms, cardiovascular symptoms, gonadal suppression, tumor, metabolic disease, psychiatric compromise, and other adverse reactions.

### 2.6 Statistical methods

We used Stata software (version 15.0, Stata MP, StataCorp, College Station, TX) to conduct NMA for dobby trials (including two or more experimental intervention groups with common control groups, or two control intervention groups, such as the placebo group and the standard treatment group) under the framework of frequency. We calculated the surface under the cumulative ranking area (SUCRA) of each intervention, summarized and ranked the differences in the remission rates of all treatment measures. The greater the ratio of SUCRA, the higher the response rate. We used node splitting methods to evaluate the difference in analysis between direct and indirect comparisons between different studies. When there was no significant statistical difference between the direct comparison and indirect comparison (*P* > 0.05), we used the consistency model for NMA, otherwise we used the inconsistency effect model. The publication bias of subgroups with more than ten studies was evaluated by observing whether the funnel plot was symmetrical. In subgroups with less than 10 studies, the Egger’s test was used [20].

### 2.7 Evidence quality assessment

We evaluated the quality of the direct comparison according to the RCT evidence quality evaluation method released by GRADE working group [13,14,15,16,17] (Table S1). At the beginning of the evaluation, all included RCTs were set at a high-quality level. They were then evaluated according to five aspects: risk of bias, inconsistency, indirectness, imprecision, and other considerations. For each criterion that a study failed to meet, the guidance level was reduced by one level. For serious non-compliance, the guidance level can be reduced by two levels. After completing the evaluation of the five projects, the final grade results were summarized.

The quality assessment of the indirect comparison was carried out according to the method described by Puhan et al. [18] and relative references [21,22,23]. First, the best comparable path was selected. The fewer the interventions present in the indirect comparison path, the higher the credibility of the results. After determining the best indirect comparison path, the evidence quality of a single direct comparison in the path was evaluated according to the method. The lowest level of evidence was selected to reflect the final evidence quality of this group of indirect comparisons. If there are both direct and indirect effects in a set of comparisons, the two comparisons are evaluated separately, and a higher level of evidence was selected to reflect the result. The final step was to assess the inconsistency of results, including baseline characteristics, common references, and differences in outcome measurements between different groups. If the difference is significant, the final quality level will be further reduced by one level. This work was carried out on GRADE profile.

### 2.8 Patient and public involvement

Our study was a meta-analysis, so we did not involve any patients in this study. Since our data come from previously published clinical data, we were unable to disseminate the results to participants.

## 3 Results

**Figure 1:**
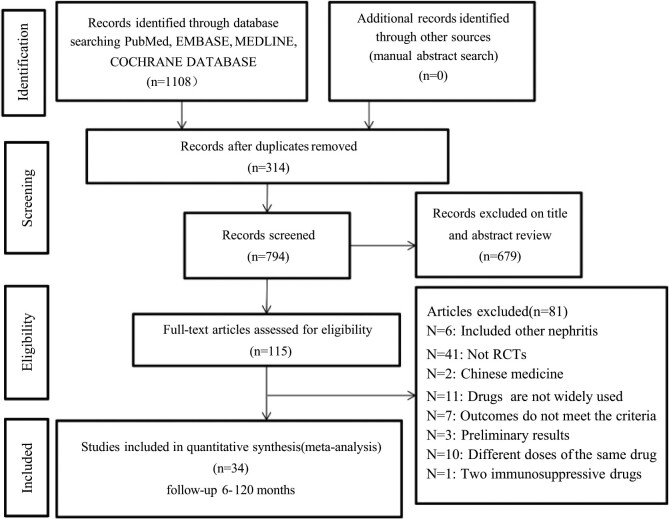
Flow chart depicting the process of identification of studies.

Using the above retrieval strategy, a total of 1,108 articles were retrieved (Table S2 for the complete retrieval strategies). After excluding duplicate and other irrelevant articles, 115 articles were suitable for analysis. After carefully reviewing each article according to the above inclusion and exclusion criteria, a total of 34 articles [9,10,11,12,24,25,26,27,28,29,30,31,32,33,34,35,36,37,38,39,40,41,42,43,44,45,46,47,48,49,50,51,52,53] were included. Of the 81 excluded articles, the following issues were found: 6 articles included other types of nephritis; 2 papers were on traditional Chinese medicine; 41 papers were not RCTs; 11 papers used uncommon drugs; the results of 7 papers did not meet the standard; 3 papers used the preliminary results; 10 papers compared different dosages of the same drug; and the outcome indicators of 1 paper did not meet the inclusion criteria. The details are shown in Figure 1.

### 3.1 Characteristics and quality of included studies

Tables S3 and S4 summarize the basic characteristics of the study, including 34 RCTs, with a total sample size of 2,064 and a follow-up period of 6–120 months. All studies reported remission rates, except that by Kosmadakis et al. [49]. All other studies reported the incidence of various side effects in detail. Of the 34 studies included, 32 [9,10,11,12,24,25,26,27,28,29,30,32,33,34,35,36,37,38,39,40,41,42,43,44,45,46,47,48,50,51,52,53] were two-arm studies and the remaining two were three-arm studies.

**Figure 2:**
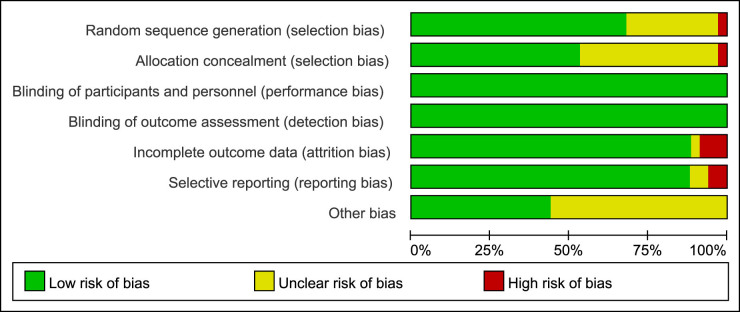
Risk of bias assessment: overall risk of bias for all included trials.

**Figure 3:**
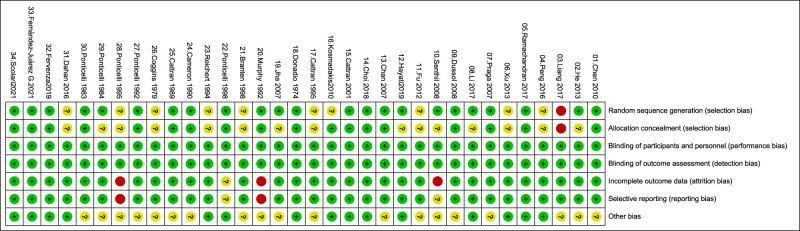
Risk of bias summary: overall risk of bias for all included trials.

The literature quality assessment is based on the Cochrane bias risk assessment tool. The overall quality is the risk of medium and low bias. Since remission rates and side effects are objective indicators, single-blind or non-blind studies have little impact on these results. Details of the literature quality assessment are shown in Figures 2 and 3 and Table S5.

### 3.2 Direct meta-analysis

#### 3.2.1 CR rate

The meta-analysis of the direct comparison showed that the curative effect of RTX was significantly inferior to that of CTX (OR 0.37, CI 0.18, 0.75) with moderate evidence. CTX was significantly better than that of conservative treatment (OR 6.26, CI 1.02, 38.45) with low evidence. CHL was significantly better than that of conservative treatment (OR 8.43, CI 3.49, 20.38), with high evidence (Figure S1).

#### 3.2.2 TR rate

A meta-analysis of the direct comparison showed that in terms of TR rate, CTX (OR 4.06, CI 2.01, 18.19) and CHL (OR 4.65, CI 2.49, 8.68) were significantly better than conservative treatment with high evidence. RTX was also significantly better than CSA (OR 6.00, CI 2.74, 13.15) with moderate evidence. STE was inferior to CHL (OR 0.38, CI 0.16, 0.87) with low evidence (Figure S2).

### 3.3 NMA

#### 3.3.1 CR rate

**Figure 4:**
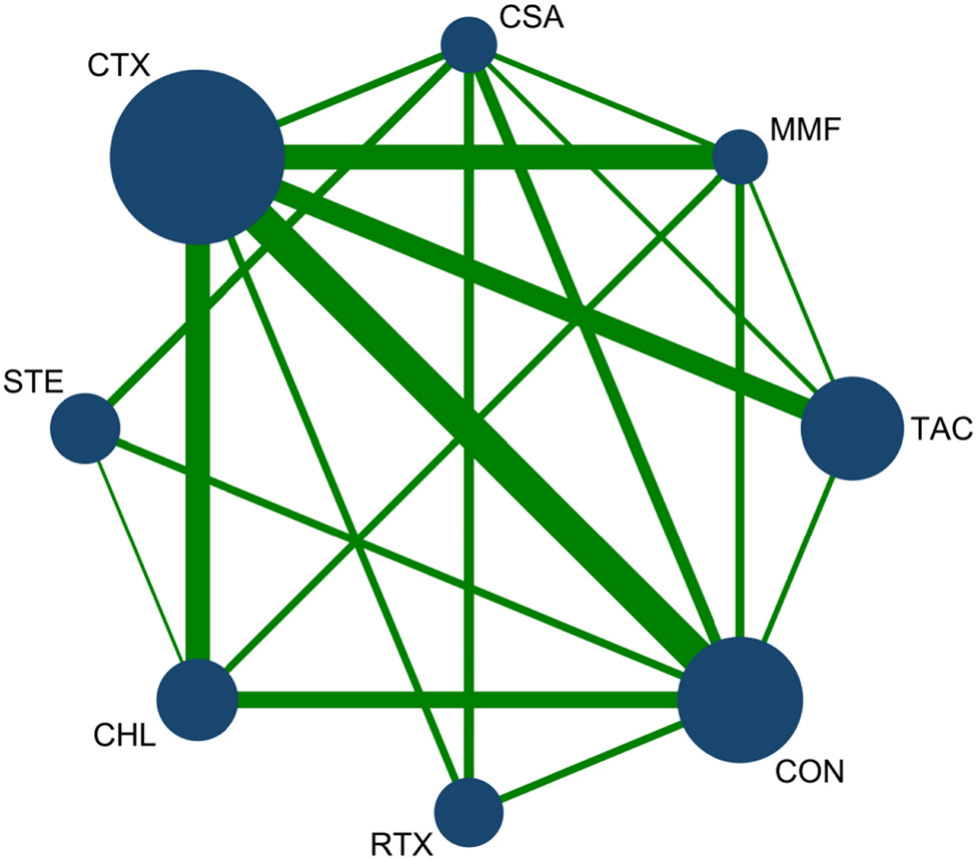
Network diagram for CR: The network of all treatments used for the evaluation of CR. The node size was found to be proportional to the number of patients who were randomized for each modality and the line thickness was corresponded to the number of direct comparisons. For example, the size of the CTX circle was the largest, while the line between CTX and CON was the thickest. This indicates that CTX had the greatest number of studies, and direct comparisons between CTX and CON were commonly found in the literature.

A total of 31 articles reported the CR rate. Among them, 18 articles reported CTX, 11 mentioned conservative treatment, 8 papers used CHL, 4 articles reported STE, 5 papers reported CSA, 8 articles mentioned TAC, 7 studies reported MMF, and 4 was RTX. The network diagram of each treatment for CR is shown in Figure 4.

##### 3.3.1.1 Publication bias

**Figure 5:**
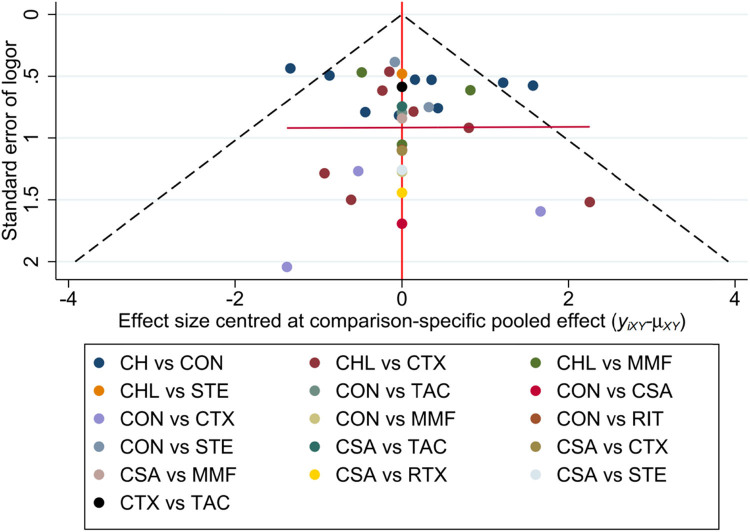
Funnel plot for CR, with a complex evidence network including 16 sets of head-to-head randomized trials as shown above. Single markers represented the individual primary studies, while the orange vertical line showed the summary effect estimate, and the dashed oblique lines showed the 95% CIs at varying degrees of precision.

A general funnel plot generated by Stata is included (Figure 5). The results showed that the included studies had no publication bias. Egger’s test was used to detect the publication bias for each direct comparison with more than two studies. If *P* > 0.1, there was no publication bias, otherwise it means publication bias (Table S6).

##### 3.3.1.2 Contribution chart

The direct comparison weights of the top three were STE vs conservative therapy (CON) (54.0%), MMF vs CTX (42.9%), and CTX vs RTX (42.0%). This means that the reliability of the above results is relatively high (Figure S3).

##### 3.3.1.3 Inconsistency test

The node splitting method was used to perform the inconsistency test. If *P* > 0.05, there was no inconsistency between these studies, otherwise there was local comparison inconsistency. The results showed that, as shown in Table S7, there was no inconsistency in all comparisons between these groups except CSA vs CTX and RTX vs CTX. First, the inconsistency model was used for NMA to evaluate the global comparison inconsistency. The results of the inconsistency model suggest that there was no inconsistency among the design effects, so it can be simplified as the consistency effect model for calculation.

##### 3.3.1.4 Results

**Figure 6:**
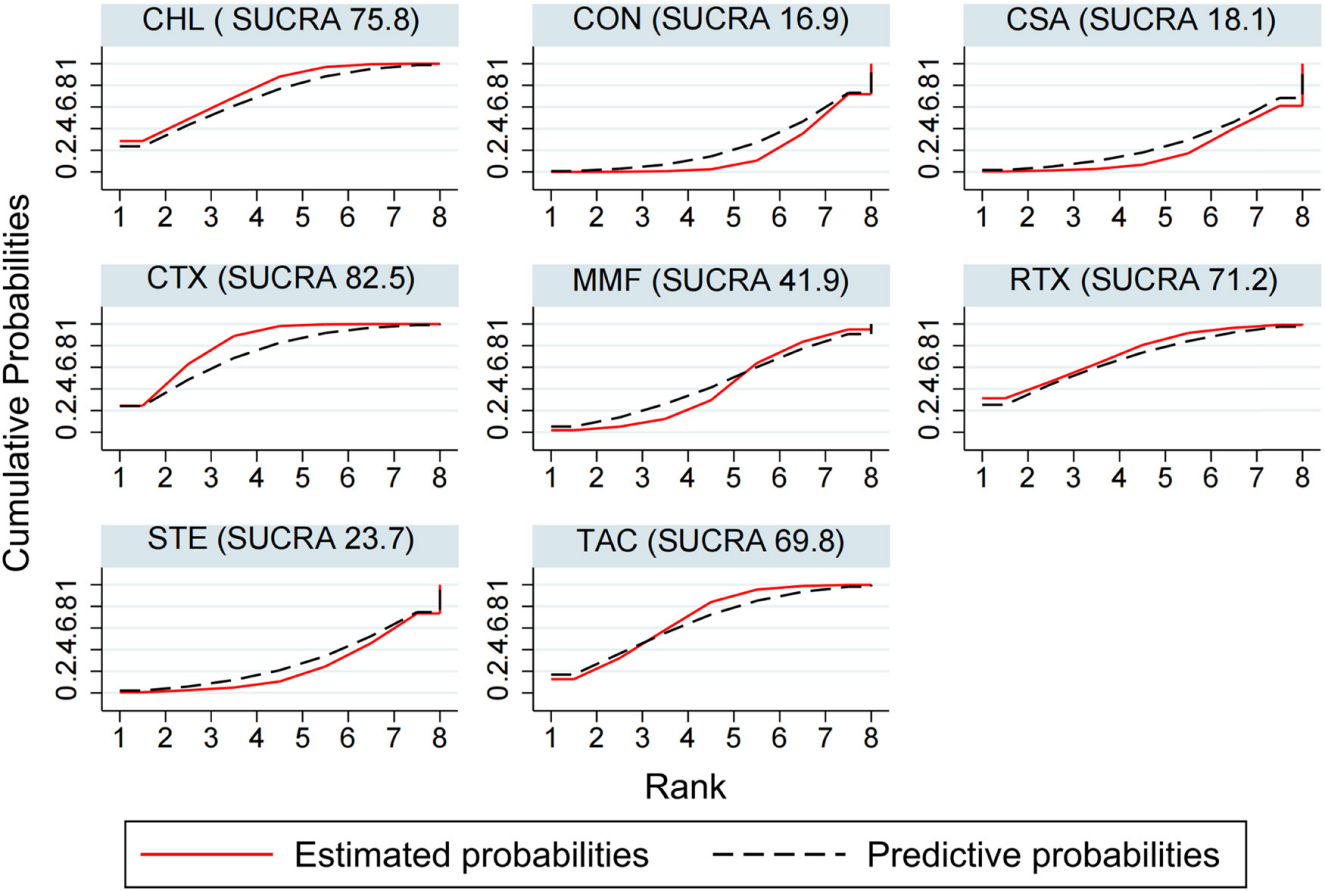
CR ranking among different interventions. A simple numerical summary to present the graphical display of cumulative ranking was used to estimate the SUCRA line for each treatment. SUCRA would be 100% when a treatment was certain to be the best and 0 when a treatment was certain to be the worst. If a treatment always ranks first, then it will have 100% SUCRA, and if it always ranks last, it will have 0 SUCRA. This enabled us to rank the treatments overall. For example, treatment CTX emerged as the best, followed by CHL, and last came CON.

The results of NMA using the consistency model suggest that for the CR rate, the ranking of SUCRA is CTX (82.5) > CHL (75.8) > RTX (71.2) > TAC (69.8) > MMF (41.9) > STE (23.7) > CSA (18.1) > CON (16.9) (Figure 6). CTX was significantly better than conservative treatment (OR 3.85; CI 1.39, 11.11) with moderate evidence. CHL was also significantly better than conservative treatment (OR 3.51; CI 1.34, 9.21), and the evidence was high. CSA was not as effective as CTX (OR 0.26, CI 0.07, 0.91) with low evidence (Table 1 and Table S8).

**Table 1:** Final results and GRADE assessment for CR

	Direct estimates		Indirect estimates		Network meta-analysis	
Pharmacological intervention	OR（95% CI）	Quality of evidence	OR（95% CI）	Quality of evidence	OR(95% CI)	Quality of evidence
**Compared with TAC**						
CTX	1.07 (0.73, 1.57)	Very low	1.08 (0.48, 2.42)	Low	1.24 (0.60, 2.57)	Low
CON	2.04 (0.43, 9.70)	Low	0.24 (0.08, 0.70)	Low	0.32 (0.10, 1.00)	Low
MMF	0.22 (0.07, 0.69)	Moderate	0.62 (0.20, 1.97)	Very low	0.58 (0.19, 1.72)	Moderate
CHL	N/A		0.80 (0.29, 2.24)	Very low	1.13 (0.35, 3.72)	Very low
CSA	0.64 (0.15, 2.76)	Low	0.35 (0.09, 1.30)	Very low	0.32 (0.09, 1.18)	Low
STE	N/A		0.22 (0.07, 0.74)	Low	0.35 (0.08, 1.51)	Low
RTX	N/A		1.55(0.17, 13.70)	Low	1.12 (0.28, 4.45)	Low
**Compared with CTX**						
CON	0.16 (0.03, 0.98)	Low	0.22(0.09, 0.55)	Moderate	0.26 (0.09, 0.72)	Moderate
MMF	0.87 (0.40, 1.90)	Moderate	0.58(0.24, 1.38)	Low	0.46 (0.18, 1.21)	Moderate
CHL	0.47 (0.21, 1.03)	Moderate	0.75(0.37, 1.54)	Low	0.91 (0.32, 2.58)	Moderate
CSA	0.11 (0.01, 1.34)	Low	0.32(0.10, 1.02)	Low	0.26 (0.07, 0.91)	Low
STE	N/A		0.21 (0.07, 0.60)	Low	0.29 (0.07, 1.11)	Low
RTX	0.37 (0.18, 0.75)	Moderate	1.44(0.19, 10.86)	Low	0.90 (0.27, 3.03)	Moderate
**Compared with CON**						
MMF	0.42 (0.03, 5.06)	Low	2.69 (0.80, 9.02)	Low	1.78 (0.52, 6.08)	Low
CHL	8.43 (3.49, 20.38)	High	3.53 (1.27, 9.80)	Low	3.51 (1.34, 9.21)	High
CSA	3.32 (0.12, 91.60)	Low	1.63 (0.55, 4.83)	Low	0.99 (0.24, 4.12)	Low
STE	0.82 (0.42, 1.60)	Low	0.94 (0.47, 1.88)	Low	1.10 (0.36, 3.38)	Low
RTX	8.63 ( 1.01, 74.11)	Low	4.03 (0.54, 30.15)	Low	3.46 (0.82, 14.56)	Low
**Compared with MMF**						
CHL	2.25 (0.29, 17.76)	Low	1.30(0.46, 3.65)	Moderate	1.97 (0.57, 6.84)	Moderate
CSA	0.85 (0.16, 4.43)	Low	0.56 (0.16, 1.94)	Low	0.56 (0.14, 2.19)	Low
STE	N/A		0.36 (0.10, 1.26)	Low	0.62 (0.14, 2.80)	Low
RTX	N/A		2.49 (0.30, 20.91)	Moderate	1.94 (0.43, 8.71)	Moderate
**Compared with CHL**						
CSA	N/A		0.43 (0.12, 1.50)	Low	0.28 (0.06, 1.24)	Low
STE	0.60 (0.23, 1.53)	Low	0.27(0.09, 0.82)	Low	0.31 (0.09, 1.08)	Low
RTX	N/A		1.92 (0.23, 15.68)	Moderate	0.99 (0.22, 4.47)	Moderate
**Compared with CSA**						
STE	0.59 (0.05, 6.96)	Low	0.64 (0.19, 2.13)	Low	1.11 (0.22, 5.48)	Low
RTX	72.44 (4.28, 1224.53)	Moderate	4.47 (0.63, 31.93)	Low	3.50 (0.70, 17.51)	Moderate
**Compared wit**h **STE**						
RTX	N/A		4.29 (0.56, 32.91)	Low	3.15 (0.57, 17.51)	Low

#### 3.3.2 TR rate

**Figure 7:**
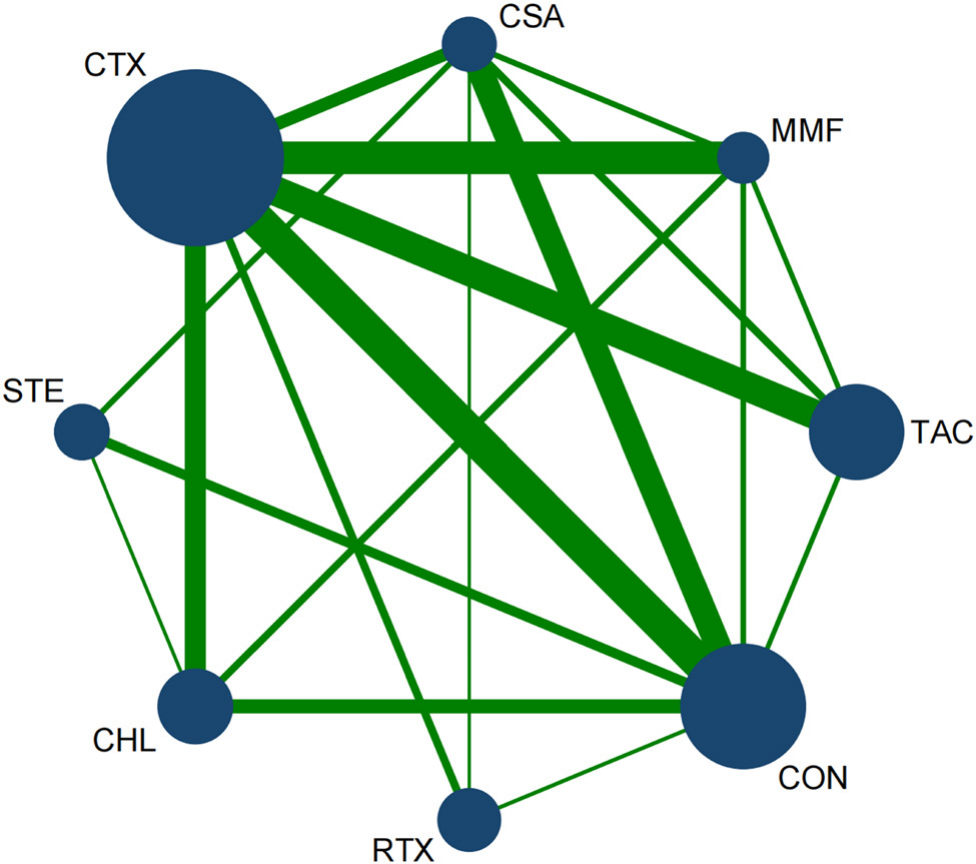
Network diagram for TR: The network of all treatments used for the evaluation of TR. The node size was found to be proportional to the number of patients who were randomized for each modality and the line thickness was corresponded to the number of direct comparisons. For example, the size of the CTX circle was the largest, while the line between CTX and CON was the thickest. This indicates that that CTX had the greatest number of studies, and direct comparisons between CTX and CON were commonly found in the literature.

A total of 33 articles reported the overall remission rate of proteinuria. Among them, 18 articles reported CTX, 13 mentioned conservative treatment, 8 papers used CHL, 4 articles reported STE, 6 papers reported CSA, 8 articles mentioned TAC, 7 researchers reported MMF, and 4 was RTX. Figure 7 shows the network diagram and consistency test results of various treatment interventions for TR.

##### 3.3.2.1 Publication bias

**Figure 8:**
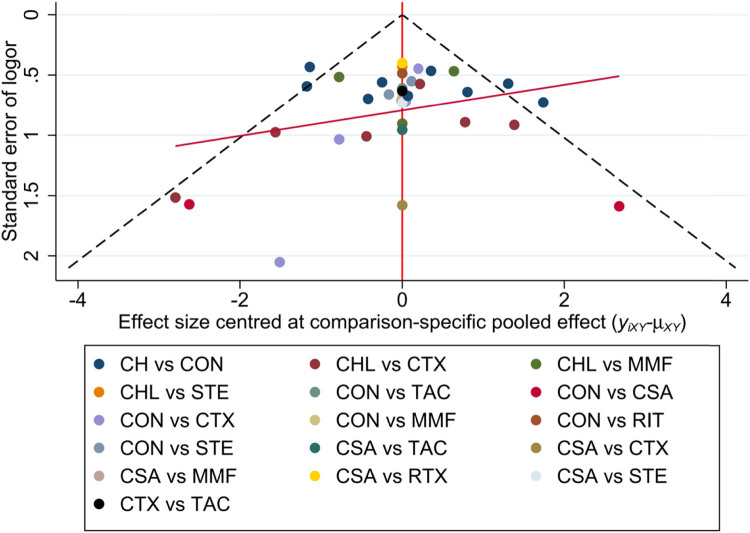
Funnel plot for TR with a complex evidence network including 16 sets of head-to-head randomized trials as shown above. Single markers represented the individual primary studies, while the orange vertical line showed the summary effect estimate, and the dashed oblique lines showed the 95% CIs at varying degrees of precision.

A general funnel plot performed by Stata is included (Figure 8). The results showed that the included studies had no publication bias. The Beggar’s and Egger’s tests were used to detect the publication bias for each direct comparison with more than two studies. If *P* > 0.1, there was no publication bias, otherwise, it means publication bias (Table S9).

##### 3.3.2.2 Contribution chart

The direct comparison weights of the top three were CHL vs CON (44.6%), CSA vs RTX (43.2%), and CTX vs CON (36.2%). This means that the reliability of the above results is relatively high (Figure S4).

##### 3.3.2.3 Inconsistency tests

Stata was used to perform the inconsistency test. If *P* > 0.05, there was no inconsistency between these studies, otherwise there is inconsistency. The results shown in Table S10 indicate no inconsistency in all comparisons between these groups, and the consistency effect model can be used.

##### 3.3.2.4 Results

**Figure 9:**
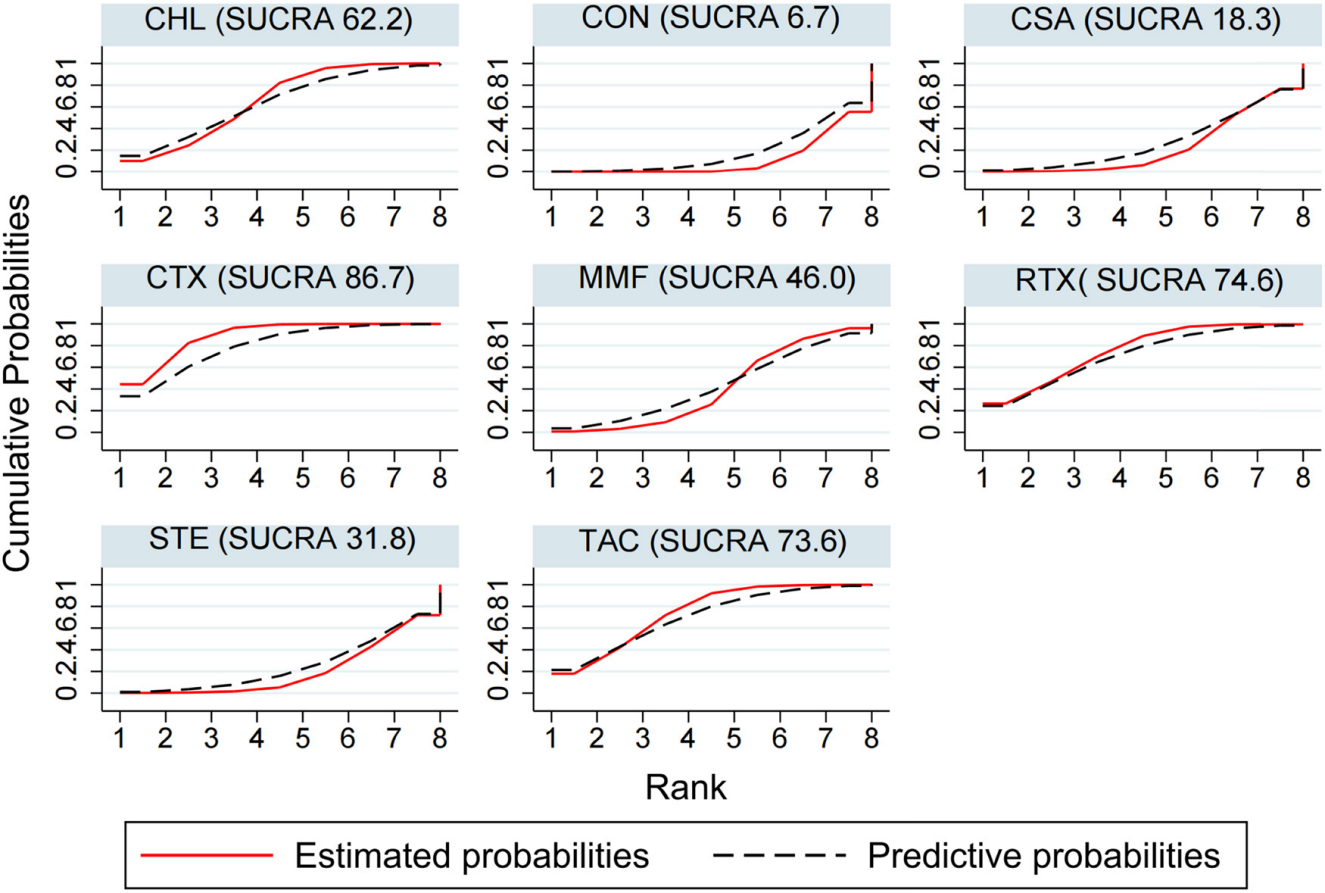
TR ranking among different interventions. A simple numerical summary to present the graphical display of cumulative ranking was used to estimate the SUCRA line for each treatment. SUCRA would be 100% when a treatment was certain to be the best and 0 when a treatment was certain to be the worst. If a treatment always ranks first, then it will have 100% SUCRA, and if it always ranks last, it will have 0 SUCRA. This enabled us to rank the treatments overall. For example, treatment CTX emerged as the best, followed by RTX, and last came CON.

The results of NMA using the consistency model suggest that for the TR rate, the ranking for SUCRA is CTX (86.7) > RTX (74.6) > TAC (73.6) > CHL (62.2) > MMF (46.0) > STE (31.8) > CSA (18.3) > CON (6.7) (Figure 9). Compared with conservative treatment, CTX (OR 4.76; CI 2.33, 9.09), CHL (OR 3.12; CI 1.45, 6.71), and RTX (OR 3.78; CI 1.41, 10.09) were associated with significantly higher probabilities of TR, with high and moderate evidence, respectively. There was no significant difference between RTX and CTX (OR 0.81, CI 0.32, 2.01) with moderate evidence; CSA was not as effective as RTX in the TR rate, the difference was statistically significant (OR 0.34, CI 0.11, 1.00), and the evidence was moderate (Table 2 and Table S11).

**Table 2:** Final results and GRADE assessment for TR

	Direct estimates		Indirect estimates		Network meta-analysis	
Pharmacological intervention	OR（95% CI）	Quality of evidence	OR（95% CI）	Quality of evidence	OR(95% (C)	Quality of evidence
**Compared with TAC**						
CTX	0.99 (0.35, 2.77)	Low	1.14 (0.47, 2.73)	Low	1.26 (0.64, 2.48)	Low
CON	0.66 (0.20, 2.17)	Low	0.23 (0.08, 0.67)	Low	0.27 (0.11, 0.65)	Low
MMF	0.47 (0.14, 1.61)	Low	0.63 (0.24, 1.71)	Low	0.52 (0.19, 1.41)	Low
CHL	N/A		0.90 (0.30, 2.76)	Low	0.84 (0.31, 2.30)	Low
CSA	0.39 (0.06, 2.55)	Low	0.47 (0.14, 1.63)	Very low	0.34 (0.11, 1.04)	Very low
STE	N/A		0.38 (0.12, 1.26)	Low	0.32 (0.09, 1.08)	Low
RTX	N/A		0.81 (0.24, 2.71)	Low	1.02 (0.34, 3.02)	Low
**Compared with CTX**						
CON	0.25 (0.12, 0.50)	High	0.20 (0.10, 0.41)	Low	0.21 (0.11, 0.43)	High
MMF	0.59 (0.19, 1.83)	Low	0.56 (0.21, 1.45)	Low	0.41 (0.17, 0.97)	Low
CHL	0.35 (0.08, 1.59)	Low	0.79 (0.36, 1.74)	High	0.67 (0.29, 1.53)	High
CSA	0.08 (0.00, 1.88)	Low	0.42 (0.12, 1.41)	Very low	0.27 (0.10, 0.75)	Very low
STE	N/A		0.34 (0.14, 0.84)	Moderate	0.25 (0.08, 0.76)	Moderate
RTX	0.56 (0.14, 2.24)	Moderate	0.72 (0.30, 1.70)	Low	0.81 (0.32, 2.01)	Moderate
**Compared with CON**						
MMF	0.83 (0.22, 3.19)	Low	2.79 (0.90, 8.70)	Low	1.92 (0.75, 4.94)	Low
CHL	4.65 (2.49, 8.68)	High	3.98 (2.25, 7.03)	Low	3.12 (1.45, 6.71)	High
CSA	0.97 (0.01, 177.01)	Very low	2.09 (0.59, 7.42)	Low	1.27 (0.45, 3.56)	Low
STE	2.15 (0.94, 4.94)	Moderate	1.69 (0.86, 3.32)	Very low	1.18 (0.45, 3.12)	Moderate
RTX	3.55 (1.37, 9.19)	Low	3.58 (1.57, 8.17)	Moderate	3.78 (1.41, 10.09)	Moderate
**Compared with MMF**						
CHL	1.50 (0.26, 8.82)	Low	1.42 (0.46, 4.39)	Low	1.62 (0.58, 4.57)	Low
CSA	0.63 (0.15, 2.54)	Low	0.75 (0.22, 2.49)	Very low	0.66 (0.21, 2.05)	Very low
STE	N/A		0.60 (0.18, 2.04)	Low	0.61 (0.18, 2.15)	Low
RTX	N/A		1.28 (0.37, 4.44)	Low	1.96 (0.61, 6.29)	Low
**Compared with CHL**						
CSA	N/A		0.53 (0.14, 1.96)	Low	0.41 (0.13, 1.29)	Low
STE	0.38 (0.16, 0.87)	Low	0.42 (0.22, 0.84)	Moderate	0.38 (0.13, 1.08)	Moderate
RTX	N/A		0.90 (0.35, 2.32)	Low	1.21 (0.39, 3.74)	Low
**Compared with CSA**						
STE	0.23 (0.06, 0.97)	Low	0.81 (0.21, 3.12）	Very Low	0.93 (0.28, 3.15)	Very Low
RTX	6.00 (2.74, 13.15)	Moderate	1.72 (0.44, 6.74）	Low	2.98 (1.00, 8.91)	Moderate
**Compared wit**h **STE**						
RTX	N/A		2.12 (0.75, 6.03）	Low	0.31 (0.09, 1.13)	Low

### 3.4 Side effect profiles

The use of immunosuppressants can reduce the immune function of patients, and the drug itself has many toxic and side effects. The adverse drug reactions analyzed in this study mainly include infection, bone marrow suppression, hepatic and renal function injury, cardiovascular events, adverse reactions of the nervous system and gastrointestinal tract, metabolic diseases, gonadal toxicity, etc. The statistics of adverse reactions of different treatments are shown in Table 3. Among the schemes, the three interventions with the highest incidence of infection were CTX (23.34%), followed by RTX (20.33%) and MMF (19.82%). The three immunosuppressive agents associated with a higher frequency of bone marrow suppression were CHL (12.9%), CTX (12.2%), and RTX (6.1%). In addition, it should be noticed that TAC was related to the highest incidence rate of glucose intolerance (17.1%) and RTX was related to the highest incidence rate of transfusion reaction (15.9%).

**Table 3:** Side effects

Side effect		Drugs							
		CTX	TAC	CSA	CHL	MMF	STE	RTX	CON
Infection	Cases	109	43	25	21	22	0	37	18
	Percentage	23.34%	17.13%	17.24%	9.68%	19.82%	0.00%	20.33%	4.16%
Leukopenia	Cases	37	1	0	25	0	0	2	0
	Percentage	7.92%	0.40%	0.00%	11.52%	0.00%	0.00%	1.10%	0.00%
Anemia	Cases	20	1	1	3	4	0	9	1
	Percentage	4.28%	0.40%	0.69%	1.38%	3.60%	0.00%	4.95%	0.23%
Hepatotoxicity	Cases	28	16	3	3	2	0	0	0
	Percentage	6.00%	6.37%	2.07%	1.38%	1.80%	0.00%	0.00%	0.00%
Hyperglycemia	Cases	35	43	3	5	2	5	3	2
	Percentage	7.49%	17.13%	2.07%	2.30%	1.80%	2.11%	1.65%	0.46%
Gastrointestinal symptoms	Cases	32	20	31	17	13	10	20	2
	Percentage	6.85%	7.97%	21.38%	7.83%	11.71%	4.22%	10.99%	0.46%
Neuropathy	Cases	29	20	36	9	4	3	52	8
	Percentage	6.21%	7.97%	24.83%	4.15%	3.60%	1.27%	28.57%	1.85%
Renal toxicity	Cases	8	10	16	0	0	0	18	2
	Percentage	1.71%	3.98%	11.03%	0.00%	0.00%	0.00%	9.89%	0.46%
Hypertension/hypotension	Cases	13	8	23	0	1	6	6	4
	Percentage	2.78%	3.19%	15.86%	0.00%	0.90%	2.53%	3.30%	0.92%
Cancer	Cases	4	0	1	2	2	1	4	1
	Percentage	0.86%	0.00%	0.69%	0.92%	1.80%	0.42%	2.20%	0.23%
Psychosis	Cases	20	0	8	2	0	5	10	1
	Percentage	4.28%	0.00%	5.52%	0.92%	0.00%	2.11%	5.49%	0.23%
Cardiovascular disease	Cases	11	3	0	0	0	6	9	6
	Percentage	2.36%	1.20%	0.00%	0.00%	0.00%	2.53%	4.95%	1.39%
Amenorrhea	Cases	5	0	0	3	0	0	0	0
	Percentage	1.07%	0.00%	0.00%	1.38%	0.00%	0.00%	0.00%	0.00%
Transfusion reaction	Cases	2	0	0	0	0	0	29	0
	Percentage	0.43%	0.00%	0.00%	0.00%	0.00%	0.00%	15.93%	0.00%

## 4 Discussion

By comparing the direct effect and mixed effect of various interventions in the treatment of IMN, this NMA draws the following conclusions. First, in the SUCRA ranking of TR and CR, CTX, TAC, RTX, and CHL were in the top four. The efficacy of these treatments was found to be significantly better than that of conservative treatment. About single interventions, the TR of RTX in the treatment of IMN was not inferior to CTX, although it is at a certain disadvantage compared to CR. Third, as one of the representative drugs of CNIs, the therapeutic effect of CSA is disappointing, so we no longer recommend it as the first-line drug for IMN treatment.

In recent years, immunosuppressive therapy of IMN has been a difficult point in the clinical practice of nephrology, which has been summarized by many studies. Ren et al. [54] completed an NMA in 2017, which included a total of 2,018 patients from 36 RCTs, and mainly observed the mortality, incidence of end-stage renal disease, and CR rate. The results showed that CTX and CHL could reduce the risk of death or deterioration of renal function, and TAC and CSA cannot protect renal function but can significantly reduce proteinuria. Zheng et al. [55] also conducted a, NMA in 2019. They included 48 RCTs, a total of 2,736 patients, including 13 immunosuppressive treatment regimens. The results showed that TAC and CTX were better than other interventions in curative effect, and the effect of the former was more significant after adding Tripterygium wilfordii glycosides. However, the side effects caused by the two should be noted, such as hyperglycemia, infection, and bone marrow suppression. Liu et al. [56] performed an NMA on the incidence of infection after immunosuppressive therapy for IMN. A total of 38 RCTs were included in the study. Statistical analysis was conducted on the infection caused by major immunosuppressive therapies. The results showed that the infection rate caused by CTX and CSA was lower than that caused by other drugs. Dai et al. [57] included a total of 4,806 patients in 75 reports (including Chinese papers) and compared the cost effect of each regimen in the treatment of IMN. The results showed that CTX was effective and cheap, TAC, although expensive, had a high remission rate. The above studies in different periods have made objective and pertinent evaluations on the selection of appropriate immunosuppressive interventions, which has certain guiding significance for the clinical treatment of IMN. However, with the continuous attention to the treatment of IMN in the past 2 years, we urgently need to reassess the above issues.

Alkylating agents, including CTX and CHL, have long been the first-line drugs for the treatment of IMN. In 2012, KDIGO guidelines took CTX as the first choice for the treatment of IMN [8], and in 2021, it was adjusted to the treatment of high-risk patients [7]. Our results suggest that CTX ranks first and CHL ranks fourth in the SUCRA ranking of TR. More importantly, in comparison with the mixed effect of conservative treatment, the TR mediated by CTX and CHL were significantly higher than that of conservative treatment, and the level of evidence was high. In addition, Howman et al. confirmed through an RCT involving 108 patients that CHL has an effect on protecting renal function [58], which is not possessed by other types of immunosuppression. In sharp contrast to the good curative effect, alkylating agents have relatively serious side effects. Our study suggests that in terms of the incidence of bone marrow suppression, CHL ranks first, CTX ranks second, and the incidence of both for infections are high. In addition, the infection combined with bone marrow suppression is more serious than the normal infection. It is worth noting that previous studies [59] have confirmed that patients who use CTX for a long time have a 3-fold increase in the risk of malignant tumor compared with normal. Infertility is another terrible side effect of CTX, especially when the cumulative dose of CTX exceeds 10 g, the incidence will increase significantly [60]. Comprehensive efficacy and side effects, we believe that CTX should be the first choice when IMN patients have severe nephrotic syndrome or renal function injury. For the above patients, if the use of CTX is contraindicated or ineffective, CHL can be used as an alternative.

RTX is an emerging drug for the treatment of IMN. It is recommended by KDIGO guidelines in 2021 for patients with moderate-risk IMN, whose main feature is a normal eGFR. As such, RTX should be the first choice [7]. RTX was first used to treat Hodgkin’s disease and is a monoclonal antibody that acts on the surface antigen CD20 of B cells. It reduces proteinuria in patients with IMN by consuming B cells [61]. Our results of NMA showed that RTX was inferior to CTX in the comparison of CR direct effect, and the difference was statistically significant. However, in the comparison of the mixed effects of CR and TR, there was no significant difference between the two drugs, and the level of evidence was moderate. In addition, compared with the conservative treatment of TR, the mixed effect of RTX was significantly higher than the former, and the difference was statistically significant with a moderate level of evidence. The efficacy of RTX was not inferior to CTX but was significantly better than conservative treatment. In terms of side effects, our study found that the incidence of complications caused by RTX, such as infusion reaction, infection, gastrointestinal syndrome, neurological symptoms, and acute renal injury, was not low. However, unlike alkylating agents, RTX rarely causes serious side effects, such as bone marrow suppression, gonadal destruction, and malignant tumors. It should be noted that we attributed the sequential treatment (TAC + RTX) used in the Starmen trial [11] to the RTX regimen. The study [33] confirmed that the relapse rate of IMN in the 24th month after treatment with CINs (TAC/CSA) was very high. The main purpose of adding RTX in this trial is to reduce the relapse rate of IMN. The final number of recurrences of IMN is only three, which confirms that RTX played a major role in this therapeutic regimen. At present, there is still a lack of RCT for RTX in the treatment of IMN. In addition, the mechanism of its efficacy has not been fully clarified and an optimal dosage has not been determined [62]. In conclusion, RTX is suitable for IMN patients who should not use CTX, such as moderate-risk patients mentioned in KDIGO guidelines in 2021.

CNIs are traditional immunosuppressive drugs for the treatment of IMN, mainly including TAC and CSA. Its effective mechanism is to reduce the production of T cell-derived lymphocytes by directly acting on renal podocytes, to eliminate proteinuria [63]. In 2021, KDIGO guidelines recommended that CNIs, like RTX, be used as the first choice for patients with moderate-risk IMN [7]. Our results show that TAC ranks second in SUCRA in TR. In the comparison of mixed effects of TR, there was no significant difference between TAC and CTX, but TAC had an obvious curative effect compared with the conservative treatment. The common side effects of TAC are hyperglycemia and infection. Properly managed, neither will cause serious harm to the patient. However, the above results of TAC need to be interpreted with caution. First, most studies regarding TAC [25,29,30,35] have a follow-up time that is less than 12 months, with absent follow-up data. This makes it impossible to objectively evaluate the relapse rate after TAC treatment. Second, the evidence level of all mixed effect results of TAC compared with other interventions is low or very low, which is not convincing enough. Therefore, the current evidence is insufficient to prove that TAC can be used as the initial treatment regimen for patients with moderate-risk IMN. CSA is another representative drug of CNIs. Our results show that in the comparison of mixed effects of TR, the efficacy of CSA is significantly worse than that of RTX. This result is due to that the included studies on CSA have been followed up for more than 2 years, and a significant increase in patients with relapse is observed at the end point of follow-up. This also confirms the view of some researchers that CNIs has a high relapse rate [33,64,65]. In view of the unsatisfactory clinical efficacy of CSA, we do not recommend it as a first-line drug for immunosuppressive therapy of IMN.

Our study also found that there were no significant differences between MMF/STE and conservative treatment in TR. As such, they should not be used as the first choice for the treatment of IMN, which was consistent with the opinions of KDIGO guidelines in 2021.

There are several limitations to this study. First, the protective effect of renal function is also an important index to measure the curative effect, which is mainly aimed at patients with renal failure. However, most of the studies included are aimed at patients with normal renal function, which makes us unable to comprehensively extract and analyze this information, so that the curative effect evaluation is not comprehensive. Second, the intervention measures included in the study were not completely unified. For example, immunosuppressive steroids delivered at different doses and routes may lead to differences in the baseline level of the included literature.

## 5 Conclusion

With regard to TR rate, CTX, TAC, RTX, and CHL were significantly better than conservative treatment. In the comparison of single regimens, the TR rate of RTX was not inferior to CTX, and the therapeutic effect of CSA was poor. Therefore, we recommend CTX and RTX as the first-line drug for IMN treatment.

## Supplementary Material

Supplementary Figure 1

Supplementary Figure 2

Supplementary Figure 3

Supplementary Figure 4

Supplementary Table 1

Supplementary Table 2

Supplementary Table 3

Supplementary Table 4

Supplementary Table 5

Supplementary Table 6

Supplementary Table 7

Supplementary Table 8

Supplementary Table 9

Supplementary Table 10

Supplementary Table 11
