# Supplementary figures and images for: Immunosuppressive treatment for idiopathic membranous nephropathy: An updated network meta-analysis
Source: Open Life Sci. 2023 Jan 10;18(1):20220527. doi: 10.1515/biol-2022-0527 (PMC9835199; doi:10.1515/biol-2022-0527)

Supplementary Figure 1. Direct meta-analysis for CR

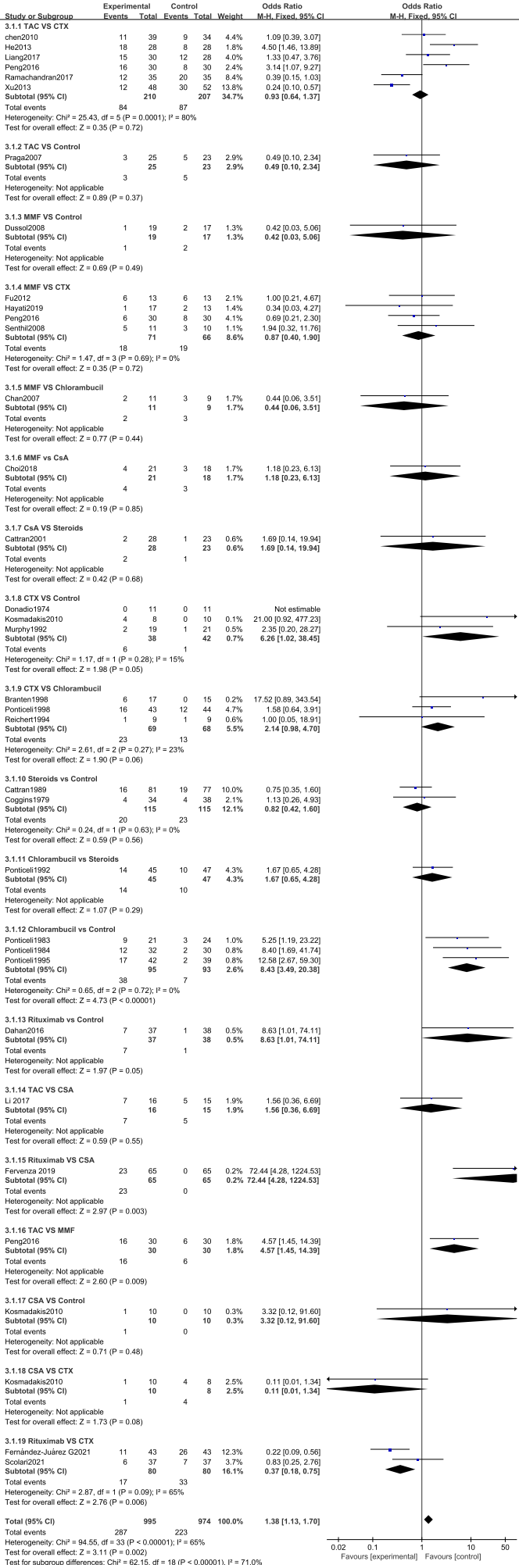

Supplement: Supplementary Figure 1 [file SupFigure_1.Direct_meta-analysis_for_CR.pdf]

Supplementary Figure 2 Direct meta-analysis for TR

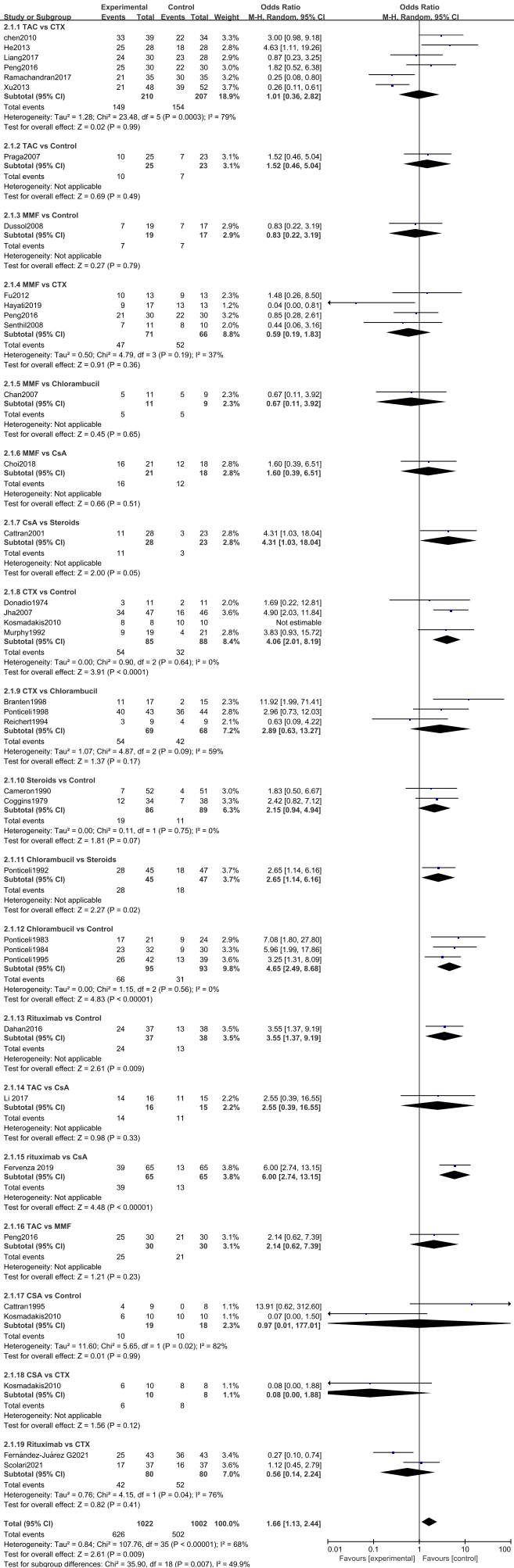

Supplement: Supplementary Figure 2 [file SupFigure_2.Direct_meta-analysis_for_TR.pdf]
