# Supplementary material for: Immunosuppressive treatment for idiopathic membranous nephropathy: An updated network meta-analysis
Source: Open Life Sci. 2023 Jan 10;18(1):20220527. doi: 10.1515/biol-2022-0527 (PMC9835199; doi:10.1515/biol-2022-0527)
Supplement: Supplementary Figure 4 [file SupFigure_4.Contribution_plots_for_TR.pdf]

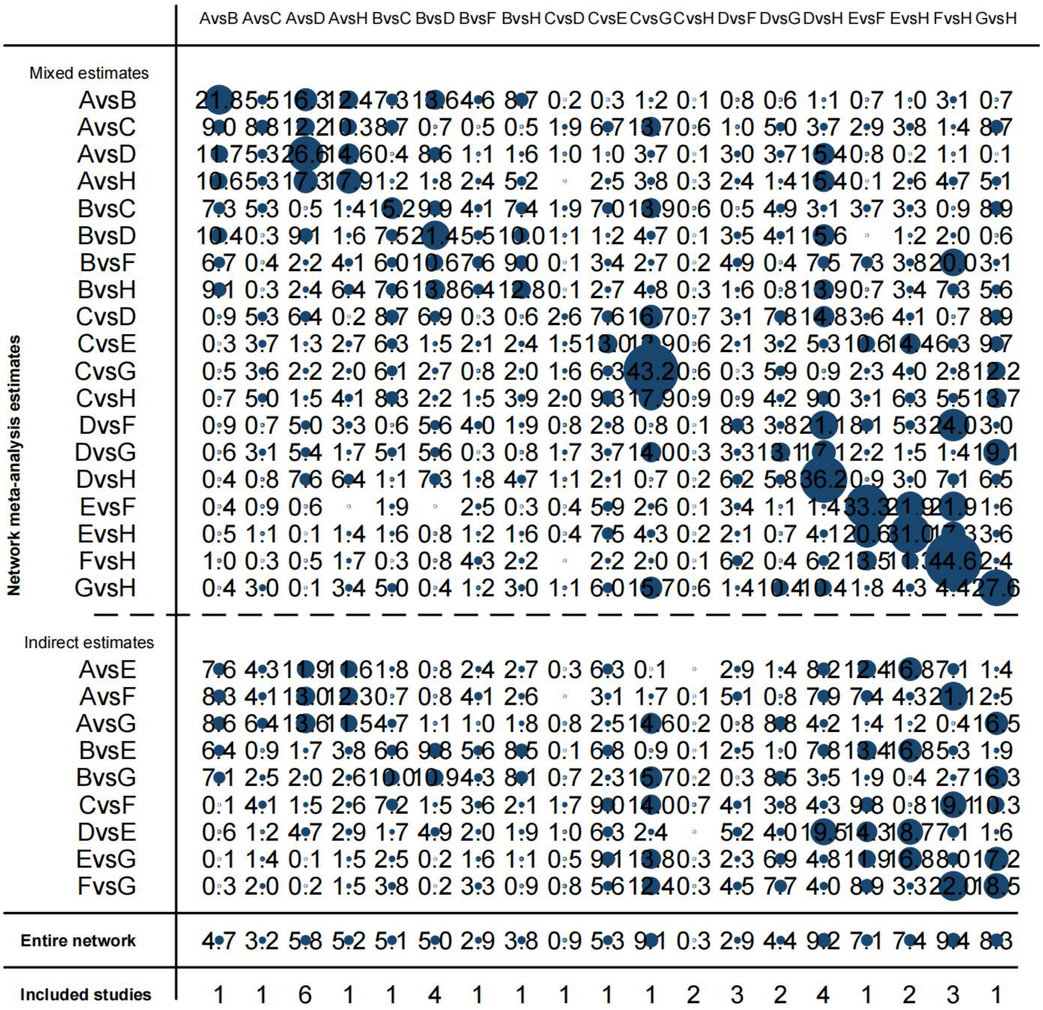

***Supplementary Figure 4**. Contribution plots for TR*

**Note：A=TAC; B=MMF; C=CSA; D=CTX; E=STE; F=CHL; G=RTX; H=CON.*
