# Supplementary material for: Immunosuppressive treatment for idiopathic membranous nephropathy: An updated network meta-analysis
Source: Open Life Sci. 2023 Jan 10;18(1):20220527. doi: 10.1515/biol-2022-0527 (PMC9835199; doi:10.1515/biol-2022-0527)
Supplement: Supplementary Table 1 [file SupTable_1.GRADE_Categories_of_Quality_of_Evidence.pdf]

***Supplementary Table 1**: GRADE Categories of Quality of Evidence*

GRADE QUALITY OF EVIDENCE	INTERPRETATION
High	Further research is VERY UNLIKELY to change our confidence in the estimate of effect
Moderate	Further research is LIKELY to have an impact on our confidence in the estimate of effect and MAY change the estimate
Low	Further research is VERY LIKELY to have an impact on our confidence in estimate of effect and is LIKELY to change the estimate
Very Low	Any estimate of effect is very uncertain

GRADE, Grading of Recommendations, Assessment, Development, and Evaluation.
