# Supplementary material for: Immunosuppressive treatment for idiopathic membranous nephropathy: An updated network meta-analysis
Source: Open Life Sci. 2023 Jan 10;18(1):20220527. doi: 10.1515/biol-2022-0527 (PMC9835199; doi:10.1515/biol-2022-0527)
Supplement: Supplementary Table 2 [file SupTable_2.Retrieval_strategies.pdf]

Supplementary Table 2 *Retrieval strategies*

PubMed

((((((((((((((((((((((((((Sendoxan[Title/Abstract]) OR (B-518[Title/Abstract])) OR (B 518[Title/Abstract])) OR (B518[Title/Abstract])) OR (Cyclophosphamide Anhydrous[Title/Abstract])) OR (Cyclophosphamide, (R)-Isomer[Title/Abstract])) OR (Cyclophosphamide, (S)-Isomer[Title/Abstract])) OR (Cytophosphane[Title/Abstract])) OR (Cyclophosphamide Monohydrate[Title/Abstract])) OR (Cytophosphan[Title/Abstract])) OR (Cytoxan[Title/Abstract])) OR (Endoxan[Title/Abstract])) OR (Neosar[Title/Abstract])) OR (NSC-26271[Title/Abstract])) OR (NSC 26271[Title/Abstract])) OR (NSC26271[Title/Abstract])) OR (Procytox[Title/Abstract])) OR ((+,-)-2-(bis(2-Chloroethyl)amino)tetrahydro-2H-1,3,2-oxazaphosphorine 2-Oxide[Title/Abstract])) OR (Monohydrate[Title/Abstract])) OR (Cyclophosphane[Title/Abstract])) OR ("Cyclophosphamide"[Mesh])) OR ((((((((((((((((Cyclosporine A[Title/Abstract]) OR (Cyclosporin A[Title/Abstract])) OR (Ciclosporin[Title/Abstract])) OR (Cyclosporin[Title/Abstract])) OR (Neoral[Title/Abstract])) OR (Sandimmun Neoral[Title/Abstract])) OR (CyA-NOF[Title/Abstract])) OR (CyA NOF[Title/Abstract])) OR (Sandimmune[Title/Abstract])) OR (Sandimmun[Title/Abstract])) OR (CsA-Neoral[Title/Abstract])) OR (CsA Neoral[Title/Abstract])) OR (CsANeoral[Title/Abstract])) OR (OL 27-400[Title/Abstract])) OR (OL 27 400[Title/Abstract])) OR (OL 27400[Title/Abstract])) OR ("Cyclosporine"[Mesh]))) OR ((((((((((((Prograf[Title/Abstract]) OR (Prograft[Title/Abstract])) OR (FR-900506[Title/Abstract])) OR (FR 900506[Title/Abstract])) OR (FR900506[Title/Abstract])) OR (Anhydrous Tacrolimus[Title/Abstract])) OR (Tacrolimus, Anhydrous[Title/Abstract])) OR (Tacrolimus Anhydrous[Title/Abstract])) OR (Anhydrous, Tacrolimus[Title/Abstract])) OR (FK-506[Title/Abstract])) OR (FK 506[Title/Abstract])) OR (FK506[Title/Abstract])) OR ("Tacrolimus"[Mesh]))) OR ((((((((((((((4-(Bis(2-chloroethyl)amino)benzenebutanoic Acid[Title/Abstract]) OR (N,N-Di-(2-chloroethyl)-p-aminophenylbutyric Acid[Title/Abstract])) OR (Chloraminophene[Title/Abstract])) OR (Chlorbutin[Title/Abstract])) OR (NSC-3088[Title/Abstract])) OR (NSC 3088[Title/Abstract])) OR (NSC3088[Title/Abstract])) OR (Leukeran[Title/Abstract])) OR (Lympholysin[Title/Abstract])) OR (Amboclorin[Title/Abstract])) OR (CB-1348[Title/Abstract])) OR (CB 1348[Title/Abstract])) OR (CB1348[Title/Abstract])) OR ("Chlorambucil"[Mesh]))) OR (((((((((CD20 Antibody, Rituximab[Title/Abstract]) OR (Rituximab CD20 Antibody[Title/Abstract])) OR (Mabthera[Title/Abstract])) OR (IDEC-C2B8 Antibody[Title/Abstract])) OR (IDEC C2B8 Antibody[Title/Abstract])) OR (IDEC-C2B8[Title/Abstract])) OR (IDEC C2B8[Title/Abstract])) OR (GP2013[Title/Abstract])) OR (Rituxan[Title/Abstract])) OR ("Rituximab"[Mesh]))) OR (((((((((((((Mycophenolate Mofetil[Title/Abstract])

OR (Mofetil, Mycophenolate[Title/Abstract])) OR (Mycophenolic Acid Morpholinoethyl Ester[Title/Abstract])) OR (Cellcept[Title/Abstract])) OR (Mycophenolate Sodium[Title/Abstract])) OR (Sodium Mycophenolate[Title/Abstract])) OR (Mycophenolate, Sodium[Title/Abstract])) OR (Myfortic[Title/Abstract])) OR (Mycophenolate Mofetil Hydrochloride[Title/Abstract])) OR (Mofetil Hydrochloride, Mycophenolate[Title/Abstract])) OR (RS 61443[Title/Abstract])) OR (RS-61443[Title/Abstract])) OR (RS61443[Title/Abstract])) OR ("Mycophenolic Acid"[Mesh]))) OR ((((Steroid[Title/Abstract]) OR (Catatoxic Steroids[Title/Abstract])) OR (Steroids, Catatoxic[Title/Abstract])) OR ("Steroids"[Mesh]))) AND ((((((((((((((((((((((Glomerulonephritides, Membranous[Title/Abstract]) OR (Membranous Glomerulonephritides[Title/Abstract])) OR (Membranous Glomerulonephritis[Title/Abstract])) OR (Nephropathy, Membranous[Title/Abstract])) OR (Membranous Glomerulopathy[Title/Abstract])) OR (Glomerulopathy, Membranous[Title/Abstract])) OR (Membranous Nephropathy[Title/Abstract])) OR (Extramembranous Glomerulopathy[Title/Abstract])) OR (Glomerulopathy, Extramembranous[Title/Abstract])) OR (Membranous Glomerulonephropathy[Title/Abstract])) OR (Glomerulonephropathy, Membranous[Title/Abstract])) OR (Idiopathic Membranous Glomerulonephritis[Title/Abstract])) OR (Glomerulonephritides, Idiopathic Membranous[Title/Abstract])) OR (Glomerulonephritis, Idiopathic Membranous[Title/Abstract])) OR (Idiopathic Membranous Glomerulonephritides[Title/Abstract])) OR (Membranous Glomerulonephritides, Idiopathic[Title/Abstract])) OR (Membranous Glomerulonephritis, Idiopathic[Title/Abstract])) OR (Idiopathic Membranous Nephropathy[Title/Abstract])) OR (Membranous Nephropathy, Idiopathic[Title/Abstract])) OR (Nephropathy, Idiopathic Membranous[Title/Abstract])) OR (Heymann Nephritis[Title/Abstract])) OR (Nephritis, Heymann[Title/Abstract])) OR ("Glomerulonephritis, Membranous"[Mesh]))) AND (((randomized controlled trial) OR (controlled clinical trial) OR (trial) OR (randomly))) **Results：295**

Embase

Session Results

....

No. Query Results	Results
#139.#25 AND #137 AND #138	**583**
#138.#48 OR #67 OR #82 OR #98 OR #110 OR #126 OR #132	2,061,170
#137.#133 OR #134 OR #135 OR #136	2,615,580
#136.'randomly'	482,463
#135.'trial'	2,322,791

#134.'controlled clinical trial' 459,694
#133.'randomized controlled trial' 894,287
#132.#127 OR #128 OR #129 OR #130 OR #131 1,795,612
#131.'steroids, catatoxic' 0
#130.'catatoxic steroids' 55
#129.'steroids' 160,268
#128.'steroid' 375,491
#127.'steroid'/exp 1,717,229
#126.#111 OR #112 OR #113 OR #114 OR #115 OR #116 OR #117 OR #118 OR #119 OR #120 OR #121 OR #122 OR #123 OR #124 OR #125 75,745
#125.'rs61443' 28
#124.'rs-61443' 311
#123.'rs 61443' 311
#122.'mycophenolate mofetil hydrochloride' 2
#121.'mycophenolate mofetil hydrochloride' 2
#120.'myfortic' 761
#119.'mycophenolate, sodium' 961
#118.'sodium mycophenolate' 60
#117.'mycophenolate sodium' 961
#116.'cellcept' 3,529
#115.'mycophenolic acid morpholinoethyl ester' 1
#114.'mofetil, mycophenolate' 155
#113.'mycophenolate mofetil' 60,355
#112.'mycophenolic acid' 49,563
#111.'mycophenolic acid'/exp 19,828
#110.#99 OR #100 OR #101 OR #102 OR #103 OR #104 OR #105 OR #106 OR #107 OR #108 OR #109 93,378
#109.'rituxan' 3,218
#108.'gp2013' 29
#107.'idec c2b8' 116
#106.'idec-c2b8' 116
#105.'idec c2b8 antibody' 2
#104.'idec-c2b8 antibody' 2
#103.'mabthera' 2,267
#102.'rituximab cd20 antibody' 2
#101.'cd20 antibody, rituximab' 829
#100.'rituximab' 93,328
#99. 'rituximab'/exp 88,445
#98. #83 OR #84 OR #85 OR #86 OR #87 OR #88 OR #89 OR #90 OR #91 OR #92 OR #93 OR #94 OR #95 OR #96 OR #97 19,894
#97. 'cb1348' 2
#96. 'cb 1348' 34

#95. 'cb-1348'	34
#94. 'amboclorin'	2
#93. 'lympholysin'	1
#92. 'leukeran'	1,106
#91. 'nsc 3088'	18
#90. 'nsc 3088'	18
#89. 'nsc-3088'	18
#88. 'chlorbutin'	29
#87. 'chloraminophene'	132
#86. 'n,n-di-(2-chloroethyl)-p-aminophenylbutyric acid'	1
#85. '4-(bis(2-chloroethyl)amino)benzenebutanoic acid'	2
#84. 'chlorambucil'	19,844
#83. 'chlorambucil'/exp	19,274
#82. #68 OR #69 OR #70 OR #71 OR #72 OR #73 OR #74 OR#75 OR #76 OR #77 OR #78 OR #79 OR #80 OR #81	91,821
#81. 'fk506'	8,112
#80. 'fk 506'	13,444
#79. 'fk-506'	13,444
#78. 'tacrolimus anhydrous'	0
#77. 'tacrolimus anhydrous'	0
#76. 'tacrolimus, anhydrous'	0
#75. 'anhydrous tacrolimus'	0
#74. 'fr900506'	4
#73. 'fr 900506'	14
#72. 'fr-900506'	14
#71. 'prograft'	298
#70. 'prograf'	3,428
#69. 'tacrolimus'	88,138
#68. 'tacrolimus'/exp	86,259
#67. #49 OR #50 OR #51 OR #52 OR #53 OR #54 OR #55 OR #56 OR #57 OR #58 OR #59 OR #60 OR #61 OR #62 OR #63 OR #64 OR #65 OR #66	166,641
#66. 'ol 27400'	1
#65. 'ol 27 400'	3
#64. 'ol 27-400'	3
#63. 'csaneoral'	0
#62. 'csa neoral'	59
#61. 'csa-neoral'	59
#60. 'sandimmun'	2,698
#59. 'sandimmune'	2,948
#58. 'cya nof'	4
#57. 'cya-nof'	4
#56. 'sandimmun neoral'	420
#55. 'neoral'	5,042
#54. 'cyclosporin'	131,664

#53. 'ciclosporin'	3,901
#52. 'cyclosporin a'	68,502
#51. 'cyclosporine a'	13,488
#50. 'cyclosporine'	161,669
#49. 'cyclosporine'/exp	157,090
#48. #26 OR #27 OR #28 OR #29 OR #30 OR #31 OR #32 OR #33 OR #34 OR #35 OR #36 OR #37 OR #38 OR #39 OR #40 OR #41 OR #42 OR #43 OR #44 OR #45 OR #46 OR #47	249,592
#47. 'cyclophosphane'	214
#46. 'monohydrate'	7,754
#45. '(+,-)-2-(bis(2-chloroethyl)amino)tetrahydro-2h-1,3,2-oxazaphosphorine 2-oxide'	9
#44. 'procytox'	80
#43. 'nsc26271'	1
#42. 'nsc 26271'	198
#41. 'nsc-26271'	198
#40. 'neosar'	201
#39. 'endoxan'	4,843
#38. 'cytoxan'	4,891
#37. 'cytophosphan'	18
#36. 'cyclophosphamide monohydrate'	30
#35. 'cytophosphane'	2
#34. 'cyclophosphamide, (s)-isomer'	0
#33. 'cyclophosphamide, (r)-isomer'	0
#32. 'cyclophosphamide anhydrous'	1
#31. 'b518'	63
#30. 'b 518'	14
#29. 'b-518'	14
#28. 'sendoxan'	107
#27. 'cyclophosphamide'	241,519
#26. 'cyclophosphamide'/exp	230,640
#25. #1 OR #2 OR #3 OR #4 OR #5 OR #6 OR #7 OR #8 OR #9 OR #10 OR #11 OR #12 OR #13 OR #14 OR #15 OR #16 OR #17 OR #18 OR #19 OR #20 OR #21 OR #22 OR #23 OR #24	10,761
#24. 'nephritis, heymann'	5
#23. 'heymann nephritis'	738
#22. 'nephropathy, idiopathic membranous'	3
#21. 'membranous nephropathy, idiopathic'	4
#20. 'idiopathic membranous nephropathy'	1,188
#19. 'membranous glomerulonephritis, idiopathic'	2
#18. 'membranous glomerulonephritides, idiopathic'	0
#17. 'idiopathic membranous glomerulonephritides'	0
#16. 'glomerulonephritis, idiopathic membranous'	3
#15. 'glomerulonephritides, idiopathic membranous'	0

#14. 'idiopathic membranous glomerulonephritis'	206
#13. 'glomerulonephropathy, membranous'	0
#12. 'membranous glomerulonephropathy'	148
#11. 'glomerulopathy, extramembranous'	0
#10. 'extramembranous glomerulopathy'	9
#9. 'membranous nephropathy'	5,611
#8. 'glomerulopathy, membranous'	6
#7. 'membranous glomerulopathy'	535
#6. 'nephropathy, membranous'	103
#5. 'membranous glomerulonephritis'	8,951
#4. 'membranous glomerulonephritides'	2
#3. 'glomerulonephritides, membranous'	0
#2. 'glomerulonephritis, membranous'	106
#1. 'membranous glomerulonephritis'/exp	8,312

Cochrane

Search Name: RCTs for IMN
Comment:

ID Search
#1 (Glomerulonephritis, Membranous):ti,ab,kw (Word variations have been searched)
#2 (Glomerulonephritides, Membranous):ti,ab,kw (Word variations have been searched)
#3 (Membranous Glomerulonephritides):ti,ab,kw (Word variations have been searched)
#4 (Membranous Glomerulonephritis):ti,ab,kw (Word variations have been searched)
#5 (Nephropathy, Membranous):ti,ab,kw (Word variations have been searched)
#6 (Membranous Glomerulopathy):ti,ab,kw (Word variations have been searched)
#7 (Glomerulopathy, Membranous):ti,ab,kw (Word variations have been searched)
#8 (Membranous Nephropathy):ti,ab,kw (Word variations have been searched)
#9 (Extramembranous Glomerulopathy):ti,ab,kw (Word variations have been searched)
#10 (Glomerulopathy, Extramembranous):ti,ab,kw (Word variations have been searched)
#11 (Membranous Glomerulonephropathy):ti,ab,kw (Word variations have been searched)
#12 (Glomerulonephropathy, Membranous):ti,ab,kw (Word variations have been searched)
#13 (Idiopathic Membranous Glomerulonephritis):ti,ab,kw (Word variations have been searched)
#14 (Glomerulonephritides, Idiopathic Membranous):ti,ab,kw (Word variations have been searched)

#15 (Glomerulonephritis, Idiopathic Membranous):ti,ab,kw (Word variations have been searched)
#16 (Idiopathic Membranous Glomerulonephritides):ti,ab,kw (Word variations have been searched)
#17 (Membranous Glomerulonephritides, Idiopathic):ti,ab,kw (Word variations have been searched)
#18 (Membranous Glomerulonephritis, Idiopathic):ti,ab,kw (Word variations have been searched)
#19 (Idiopathic Membranous Nephropathy):ti,ab,kw (Word variations have been searched)
#20 (Membranous Nephropathy, Idiopathic):ti,ab,kw (Word variations have been searched)
#21 (Nephropathy, Idiopathic Membranous):ti,ab,kw (Word variations have been searched)
#22 (Heymann Nephritis):ti,ab,kw (Word variations have been searched)
#23 (Nephritis, Heymann):ti,ab,kw (Word variations have been searched)
#24 #1 OR #2 OR #3 OR #4 OR #5 OR #6 OR #7 OR #8 OR #9 OR #10 OR #11 OR #12 OR #13 OR #14 OR #15 OR #16 OR #17 OR #18 OR #19 OR #20 OR #21 OR #22 OR #23
#25 (cyclophosphamide):ti,ab,kw (Word variations have been searched)
#26 (Sendoxan):ti,ab,kw (Word variations have been searched)
#27 (B-518):ti,ab,kw (Word variations have been searched)
#28 (B 518):ti,ab,kw (Word variations have been searched)
#29 (B518):ti,ab,kw (Word variations have been searched)
#30 (Cyclophosphamide Anhydrous):ti,ab,kw (Word variations have been searched)
#31 (Cytophosphane):ti,ab,kw (Word variations have been searched)
#32 (Cyclophosphamide Monohydrate):ti,ab,kw (Word variations have been searched)
#33 (Cytophosphan):ti,ab,kw (Word variations have been searched)
#34 (Cytoxan):ti,ab,kw (Word variations have been searched)
#35 (Endoxan):ti,ab,kw (Word variations have been searched)
#36 (Neosar):ti,ab,kw (Word variations have been searched)
#37 (NSC-26271):ti,ab,kw (Word variations have been searched)
#38 (NSC 26271):ti,ab,kw (Word variations have been searched)
#39 (NSC26271):ti,ab,kw (Word variations have been searched)
#40 (Procytox):ti,ab,kw (Word variations have been searched)
#41 (Monohydrate):ti,ab,kw (Word variations have been searched)
#42 (Cyclophosphane):ti,ab,kw (Word variations have been searched)
#43 #25 OR #26 OR #27 OR #28 OR #29 OR #30 OR #31 OR #32 OR #33 OR #34 OR #35 OR #36 OR #37 OR #38 OR #39 OR #40 OR #41 OR #42
#44 (Cyclosporine):ti,ab,kw (Word variations have been searched)
#45 (Cyclosporine A):ti,ab,kw (Word variations have been searched)
#46 (Cyclosporin A):ti,ab,kw (Word variations have been searched)
#47 (Ciclosporin):ti,ab,kw (Word variations have been searched)
#48 (Cyclosporin):ti,ab,kw (Word variations have been searched)

#49 (Neoral):ti,ab,kw (Word variations have been searched)
#50 (Sandimmun Neoral):ti,ab,kw (Word variations have been searched)
#51 (CyA-NOF):ti,ab,kw (Word variations have been searched)
#52 (CyA NOF):ti,ab,kw (Word variations have been searched)
#53 (Sandimmune):ti,ab,kw (Word variations have been searched)
#54 (Sandimmun):ti,ab,kw (Word variations have been searched)
#55 (CsA-Neoral):ti,ab,kw (Word variations have been searched)
#56 (CsA Neoral):ti,ab,kw (Word variations have been searched)
#57 (CsANeoral):ti,ab,kw (Word variations have been searched)
#58 (OL 27 400):ti,ab,kw (Word variations have been searched)
#59 (OL 27400):ti,ab,kw (Word variations have been searched)
#60 #44 OR #45 OR #46 OR #47 OR #48 OR #49 OR #50 OR #51 OR #52 OR #53 OR #54 OR #55 OR #56 OR #57 OR #58 OR #59
#61 (Tacrolimus):ti,ab,kw (Word variations have been searched)
#62 (Prograf):ti,ab,kw (Word variations have been searched)
#63 (Prograft):ti,ab,kw (Word variations have been searched)
#64 (FR-900506):ti,ab,kw (Word variations have been searched)
#65 (FR 900506):ti,ab,kw (Word variations have been searched)
#66 (FR900506):ti,ab,kw (Word variations have been searched)
#67 (Anhydrous Tacrolimus):ti,ab,kw (Word variations have been searched)
#68 (Tacrolimus, Anhydrous):ti,ab,kw (Word variations have been searched)
#69 (Tacrolimus Anhydrous):ti,ab,kw (Word variations have been searched)
#70 (Anhydrous, Tacrolimus):ti,ab,kw (Word variations have been searched)
#71 (FK-506):ti,ab,kw (Word variations have been searched)
#72 (FK 506):ti,ab,kw (Word variations have been searched)
#73 (FK506):ti,ab,kw (Word variations have been searched)
#74 #61 OR #62 OR #63 OR #64 OR #65 OR #66 OR #67 OR #68 OR #69 OR #70 OR #71 OR #72 OR #73
#75 (Chlorambucil):ti,ab,kw (Word variations have been searched)
#76 (Chloraminophene):ti,ab,kw (Word variations have been searched)
#77 (Chlorbutin):ti,ab,kw (Word variations have been searched)
#78 (NSC-3088):ti,ab,kw (Word variations have been searched)
#79 (NSC 3088):ti,ab,kw (Word variations have been searched)
#80 (NSC3088):ti,ab,kw (Word variations have been searched)
#81 (Leukeran):ti,ab,kw (Word variations have been searched)
#82 (Lympholysin):ti,ab,kw (Word variations have been searched)
#83 (Amboclorin):ti,ab,kw (Word variations have been searched)
#84 (CB-1348):ti,ab,kw (Word variations have been searched)
#85 (CB 1348):ti,ab,kw (Word variations have been searched)
#86 (CB1348):ti,ab,kw (Word variations have been searched)
#87 #75 OR #76 OR #77 OR #78 OR #79 OR #80 OR #81 OR #82 OR #83 OR #84 OR #85 OR #86
#88 (Rituximab):ti,ab,kw (Word variations have been searched)
#89 (CD20 Antibody, Rituximab):ti,ab,kw (Word variations have been searched)

#90 (Rituximab CD20 Antibody):ti,ab,kw (Word variations have been searched)
#91 (Mabthera):ti,ab,kw (Word variations have been searched)
#92 (IDEC-C2B8 Antibody):ti,ab,kw (Word variations have been searched)
#93 (IDEC C2B8 Antibody):ti,ab,kw (Word variations have been searched)
#94 (IDEC-C2B8):ti,ab,kw (Word variations have been searched)
#95 (IDEC C2B8):ti,ab,kw (Word variations have been searched)
#96 (GP2013):ti,ab,kw (Word variations have been searched)
#97 (Rituxan):ti,ab,kw (Word variations have been searched)
#98 #88 OR #89 OR #90 OR #91 OR #92 OR #93 OR #94 OR #95 OR #96 OR #97
#99 (Mycophenolic Acid):ti,ab,kw (Word variations have been searched)
#100 (Mycophenolate Mofetil):ti,ab,kw (Word variations have been searched)
#101 (Mofetil, Mycophenolate):ti,ab,kw (Word variations have been searched)
#102 (Mycophenolic Acid Morpholinoethyl Ester):ti,ab,kw (Word variations have been searched)
#103 (Cellcept):ti,ab,kw (Word variations have been searched)
#104 (Mycophenolate Sodium):ti,ab,kw (Word variations have been searched)
#105 (Sodium Mycophenolate):ti,ab,kw (Word variations have been searched)
#106 (Mycophenolate, Sodium):ti,ab,kw (Word variations have been searched)
#107 (Myfortic):ti,ab,kw (Word variations have been searched)
#108 (Mycophenolate Mofetil Hydrochloride):ti,ab,kw (Word variations have been searched)
#109 (Mofetil Hydrochloride, Mycophenolate):ti,ab,kw (Word variations have been searched)
#110 (RS 61443):ti,ab,kw (Word variations have been searched)
#111 (RS-61443):ti,ab,kw (Word variations have been searched)
#112 (RS61443):ti,ab,kw (Word variations have been searched)
#113 #99 OR #100 OR #101 OR #102 OR #103 OR #104 OR #105 OR #106 OR #107 OR #108 OR #109 OR #110 OR #111 OR #112
#114 (Steroids):ti,ab,kw (Word variations have been searched)
#115 (Steroid):ti,ab,kw (Word variations have been searched)
#116 (Catatoxic Steroids):ti,ab,kw (Word variations have been searched)
#117 (Steroids, Catatoxic):ti,ab,kw (Word variations have been searched)
#118 #114 OR #115 OR #116 OR #117
#119 (randomized controlled trial):ti,ab,kw (Word variations have been searched)
#120 (controlled clinical trial):ti,ab,kw (Word variations have been searched)
#121 (random):ti,ab,kw (Word variations have been searched)
#122 (control):ti,ab,kw (Word variations have been searched)
#123 (trial):ti,ab,kw (Word variations have been searched)
#124 #119 OR #120 OR #121 OR #122 OR #123
#125 #43 OR #60 OR #74 OR #87 OR #98 OR #113 OR #118
#126 #24 AND #124 AND #125 **231**
