# Supplementary material for: Immunosuppressive treatment for idiopathic membranous nephropathy: An updated network meta-analysis
Source: Open Life Sci. 2023 Jan 10;18(1):20220527. doi: 10.1515/biol-2022-0527 (PMC9835199; doi:10.1515/biol-2022-0527)
Supplement: Supplementary Table 3 [file SupTable_3.Baseline_of_patients_in_the_RCTs.pdf]

Supplementary Table 3. *Baseline characteristics of patients in the RCTs*

First author	Year	Nation	Follow-up time	Mean Age(y)	Gender (M/F)	Sample	Histology grading of IMN	Urine Protein (g/24hr)	Albumin (g/L)	Scr (umol/L)
**TAC vs CTX**										
Chen	2010	China	12	T:47.2<br>C:48.6	T:23/16<br>C:18/16	39/34	T:16/21/2/0<br>C:16/17/1/0	T:7.71<br>C:7.28	T:23.1<br>C:23.1	T:75.7<br>C:85.0
He	2013	China	12	T:45.4<br>C:47.2	T:20/8<br>C:19/9	28/28	T:12/9/5/2<br>C:13/11/3/1	T:6.76<br>C:6.38	T:19.8<br>C:20.6	T:81.56<br>C:82.45
Liang	2017	China	12	T:48.2<br>C:53.9	T:16/14<br>C:9/19	30/28	T:20/9/1/0<br>C:19/9/1/0	T:5.9<br>C:6.9	T:26.5<br>C:24.1	T:70.7<br>C:81.0
Peng	2016	China	9	A:43.9<br>B:40.8<br>C:39.9	A:17/13<br>B:16/14<br>C:14/16	A:30<br>B:30<br>C:30	A:10/15/5/0<br>B:10/13/7/0<br>C:12/13/5/0	A:11.7<br>B:12.9<br>C:12.1	A:20.5<br>B:19.8<br>C:21.9	A:82.4<br>B:78.4<br>C:78.7
Ramachandran	2017	India	24	T:38.66<br>C:40.80	T:27/8<br>C:20/15	35/35	NR	T:6.76<br>C:5.44	T:22.0<br>C:22.3	T:79.56<br>C:80.44
Xu	2013	China	18	T:56.3<br>C:57.8	T:31/17<br>C:30/22	48/52	T:9/31/7/1<br>C:5/35/11/7	T:5.39<br>C:5.10	T:18.4<br>C:19.3	T:77.5<br>C:87.7
**TAC vs Control**										
Praga	2007	Spain	30	T:43.7<br>C:50.1	T:20/5<br>C:20/3	25/23	T:4/15/3/0<br>C:4/18/1/0	T:7.2<br>C:8.4	T:27<br>C:29	T:86.6<br>C:97.2
**TAC vs CsA**										
Li	2017	China	6	T:39.4<br>C:42.8	T:12/4<br>C:13/2	16/15	T:1/11/4/0<br>C:3/11/1/0	T:9.5<br>C:9.7	T:22.8<br>C:23.2	T:71.8<br>C:73.3
**MMF vs Control**										
Dussol	2008	France	12	T:47.8<br>C:55.9	T:17/2<br>C:15/2	19/17	T:13/6/0/0<br>C:8/9/0/0	T:6.2<br>C:9.5	T:23.2<br>C:20.2	T:89.3<br>C:96.4
**MMF vs CTX**										
Senthil	2008	India	12	NR	NR	11/10	NR	NR	NR	NR
Fu	2012	China	36	T:43.1<br>C:42.7	T:9/4<br>C:8/5	13/13	T:1/4/5/3<br>C:2/3/6/2	T:9.57<br>C:9.42	T:23.4<br>C:23.5	T:68.8<br>C:66.3
Hayati	2019	Iran	6	T:38.11<br>C:38.69	T:10/7<br>C:9/4	17/13	NR	T:5.2<br>C:8.8	NR	NR
**MMF vs Chlorambucil**										
Chan	2007	China	15	49.5	13/7	11/9	NR	5.7	26.5	95.1
**MMF vs CsA**										
Choi	2018	SouthKorea	11.2	T:57.7<br>C:52.7	T:16/5<br>C:9/9	21/18	NR	T:8.9<br>C:8.4	T:23<br>C:25	T:97.2<br>C:79.6
**CsA vs Steroids**										
Cattran	2001	Canada	18	T:47<br>C:49	T:26/2<br>C:16/7	28/23	NR	T:9.7<br>C:8.8	T:28<br>C:27	T:114.9<br>C:97.8
**CsA vs CTX vs Control**										
Kosmadakis	2010	Greece	9	A:50.5<br>B:55.4	A:8/2<br>B:4/4	A:10<br>B:8	NR	A:6.6<br>B:7.0	A:27<br>B:28	NR

				C:51.8	C:5/5	C:10		C:5.2	C:22	
**CsA vs Control**										
Cattran	1995	Canada	12	T:44<br>C:40	T:8/1<br>C:6/2	9/8	NR	T:11.5<br>C:12.8	T:29<br>C:30	T:186<br>C:204
**CTX vs Control**										
Donadio	1974	USA	12	T:42.6<br>C:45.8	T:9/2<br>C:8/3	11/11	NR	T:7.8<br>C:7.6	T:27<br>C:23	T:106.1<br>C:97.2
Jha	2007	India	120	T:38.0<br>C:37.2	T:30/17<br>C:27/19	47/46	NR	T:6.11<br>C:5.91	T:23.4<br>C:24.2	T:107.0<br>C:103.4
Murphy	1992	Australia	24	T:47<br>C:40	T:12/7<br>C:14/7	19/21	T:4/14/1/0<br>C:5/15/1/0	T:5.0<br>C:3.9	T:28<br>C:30	T:110<br>C:90
**CTX vs Chlorambucil**										
Branten	1998	Netherlands	T:26<br>C:38	T:53<br>C:51	T:15/2<br>C:15/0	17/15	NR	T:11<br>C:9	T:22<br>C:22	T:274<br>C:219
Ponticeli	1998	Italy	12	T:48<br>C:50	T:29/16<br>C:37/13	45/50	No detail	T:6.85<br>C:7.96	NR	T:91.9<br>C:93.7
Reichert	1994	Netherlands	15	T:49<br>C:45	T:8/1<br>C:9/0	9/9	NR	T:9.8<br>C:8.5	T:25.9<br>C:22.9	T:218<br>C:260
**Steroids vs Control**										
Cameron	1990	UK	36	T:45<br>C:44	T:43/9<br>C:43/8	52/51	NR	T:10.8<br>C:10.4	T:26<br>C:25	T:114<br>C:115
Cattran	1989	Canada	48	T:46<br>C:45	T:61/20<br>C:44/33	81/77	T:6/33/33/9<br>C:7/35/28/7	T:6.9<br>C:5.2	T:27<br>C:31	T:120<br>C:103
Coggins	1979	USA	23	NR	T:22/12<br>C:20/18	34/38	T:5/18/9/2<br>C:9/20/8/1	T:9.4<br>C:8.3	NR	T:97.2<br>C:88.4
**Chlorambucil vs Steroids**										
Ponticeli	1992	Italy	48	T:46<br>C:47	T:32/13<br>C:27/20	45/47	No detail	T:7.6<br>C:7.0	NR	T:88.4<br>C:88.4
**Chlorambucil vs Control**										
Ponticeli	1995	Italy	120	T:43.5<br>C:42	T:34/8<br>C:29/10	42/39	T:11/21/8/2<br>C:7/23/7/2	T:6.2<br>C:5.3	NR	T:93.8<br>C:93.1
Ponticeli	1984	Italy	T:31<br>C:37	T:42.6<br>C:44.9	T:26/6<br>C:22/8	32/30	T:10/16/4/2<br>C:7/16/6/1	NR	NR	T:93.7<br>C:95.5
Ponticeli	1983	Italy	T:28<br>C:32	T:39.4<br>C:45.4	T:18/3<br>C:19/5	21/24	NR	NR	NR	T:93.4<br>C:97.2
**Rituximab vs Control**										
Dahan	2016	France	17	T:53<br>C:58	T:28/9<br>C:24/14	37/38	NR	NR	T:22<br>C:22	T:98.1<br>C:91.1
**Rituximab vs CSA**										
Fervenza	2019	USA	24	T:51.9<br>C:52.2	T:47/18<br>C:53/12	65/65	NR	T:8.9<br>C:8.9	T:25<br>C:25	T:115<br>C:115
**Rituximab vs CTX**										
Fernández-Juárez G	2021	Spain	24	T:55.2<br>C:56.2	T:31/12<br>C:24/19	43/43	NR	T:6.7<br>C:4.8	T:26<br>C:26	T:88.4<br>C:88.4
Scolari	2021	Italy	24	T:54<br>C:55	T:28/9<br>C:25/12	37/37	T:4/25/7/1<br>C:8/16/8/2	T:6<br>C:6	T:20<br>C:20	T:88.4<br>C:88.4
