# Supplementary material for: Immunosuppressive treatment for idiopathic membranous nephropathy: An updated network meta-analysis
Source: Open Life Sci. 2023 Jan 10;18(1):20220527. doi: 10.1515/biol-2022-0527 (PMC9835199; doi:10.1515/biol-2022-0527)
Supplement: Supplementary Table 4 [file SupTable_4.Characteristics_of_patients_in_the_RCTs.pdf]

Supplementary Table 4.*Characteristics of patients in the RCTs*

First author	Year	Study design	Intervention		PR/CR	Adverse Events	Notes
			**T-Group**	**C-Group**	**Y/N**	**Y/N**	
**TAC vs CTX**							
Chen	2010	MC/NB/no placebo/RCT	**TAC** (the initial dosage was 0.1 mg/kg/d,q12h.Later doses were adjusted to achieve a whole-blood 12 hours trough level of 5-10 ng/mL in the first 6 months and 2-5 ng/mL in the next 3 months) **Prednisone**(1mg/kg/d for 4 weeks, tapered gradually, and discontinued by 8 months).	**CTX** (100 mg/d oral daily for 4 months. Dosage was reduced by 50 mg/d if the total white blood cell count fell below 4000/L) **Prednisone** (1mg/kg/d for 4 weeks, tapered gradually, and discontinued by 8 months).	Y	Y	None
He	2013	SC/NB/no placebo/RCT	**TAC**(initial dosage was 1 mg/d for 1 week. Later an alternative dosage of 1 mg one day and 2 mg the other was used. The dosage of 2 mg was divided into 2 equal doses at 12-hour intervals. Dosage was titrated according to a whole blood concentration, with a target of 2-4 ng/mL) **Prednisone** (1mg/kg/d for 4 weeks, 5 mg per 2 weeks down to a dosage of 30 mg/day).	**CTX** (dosage was 750 mg/m^2 body surface, intravenous once every 4 weeks for 24 weeks) **Prednisone** (1mg/kg/d for 4 weeks, by 5 mg per 2 weeks down to a dosage of 30 mg/day).	Y	Y	None
Liang	2017	SC/NB/no placebo/RCT	**TAC** (initial dosage was 0.05-0.1mg/kg/d,q12h. Later doses were adjusted to achieve a whole-blood 12 hours trough level of 5-10 ng/mL in the first 6 months and 2-5ng/mL in the next 3 months. Then, the dose was tapered gradually and discontinued at the end of 12 months)	**CTX** (dosage was 0.5–0.75 g/m^2 once every month for the initial 6 months and once in every 2–3 months for the later period) **Prednisone** (1mg/kg/d for 4 weeks, by 5 mg per 2 weeks down to a dosage of 30 mg/day).	Y	Y	None
Peng	2016	SC/NB/no placebo/RCT	Group A: **TAC** (initial dosage was 0.05	Group B: **CTX**(dosage was 0.75 g/m^2	Y	Y	None

			mg/kg/d,q12h. Later doses were adjusted to achieve a whole-blood 12 hours trough level 4-8 ng/mL in the first 6 months and 2-4ng/mL in the next 3 months)<br>**Prednisone** (0.5mg/kg/d for 8 weeks, then decreased by 5 mg per 2 weeks down to a dosage of 20 mg/day).	once every month for the initial 6 months and once in every 3 months for the later period)<br>**Prednisone**(1 mg/kg/d for 8 weeks, decreased by 5 mg per 2 weeks down to a dosage of 20 mg/day).			
			Group C:<br>**MMF** (1.5-2.0 g/d in two doses)<br>**Prednisone** (1 mg/kg/d for 8 weeks, decreased by 5 mg per 2 weeks down to a dosage of 20 mg/day).				
Ramachandran	2017	SC/NB/no placebo/RCT	**TAC** (initial dosage was 0.1 mg/kg/d,q12h. Later doses were adjusted to achieve a whole-blood 12 hours trough level of 5-10 ng/mL in the first 6 months and 4-8 ng/mL in the next 6 months)<br>**Prednisone** (0.5mg/kg/d for 6 months, then tapered and stopped).	Intravenous **methylprednisolone** 1 g/day in 100 mL normal saline was administered over 60 min on three consecutive days followed by oral prednisolone at 0.5 mg/kg per day for 27 days in the first, third, and fifth month and oral **CTX** at 2 mg/kg per day in the second, fourth, and sixth month	Y	Y	All the patients were continued on a maximum tolerable dose of **ACEI** or **ARBs** and statins
Xu	2013	SC/NB/no placebo/RCT	**TAC** (initial dosage was 0.1 mg/kg/d,q12h. Later doses was in accordance to the measured serum concentration)<br>**Prednisone** (initial dose was 0.5mg/kg/d , then tapered and stopped).	**CTX**(dosage was 0.5-0.75 g/m^2 once every month for the initial 6 months and once every 3 months for the later period)<br>**Prednisone** (initial dose was 1mg/kg/d, then tapered and stopped).	Y	Y	None
**TAC vs Control**							
Praga	2007	SC/NB/no placebo/RCT	**TAC** (initial dosage was 0.05mg/kg/d,q12h. Later doses were adjusted to achieve a whole-blood 12 hours trough level 3-5 ng/mL)	Renin-angiotensin blockers, statins, low-salt and low-protein diet	Y	Y	All the patients were instructed to maintain the same doses of **ACEI** or **ARB** that

							they were taking at randomization until the end of the study
**TAC vs CsA**							
Li	2017	SC/NB/no placebo/RCT	**TAC** (initial dosage was 0.05-0.1 mg/kg/d,q12h. Later doses were adjusted to achieve a whole-blood 12 hours trough level 5-10 ng/mL) **Prednisone** (initial dosage was 0.5 mg/ kg/d. Then the dosage was tapered slowly by 5 mg per month down to a dosage of 10 mg/d and maintained that dosage throughout the remainder of the 6-month therapy period).	**CsA** (initial dose was 3-5 mg/kg, and the dosage was adjusted to maintain a 100-200 ng/mL trough blood level) **Prednisone** (initial dosage was 0.5 mg/ kg/d. Then the dosage was tapered slowly by 5 mg per month down to a dosage of 10 mg/d and maintained that dosage throughout the remainder of the 6-month therapy period).	Y	Y	None
**MMF vs Control**							
Dussol	2008	MC/NB/no placebo/RCT	**MMF** (initial dose was 250 mg/d, progressively increased by 250 mg every other day to 2g/d)	Renin-angiotensin blockers, statins, low-salt and low-protein diet, and diuretics in case of edema	Y	Y	The supportive treatment was also used in the MMF group
**MMF vs CTX**							
Senthil	2008	SC/NB/no placebo/RCT	**MMF** (2.0 g/d in two doses) **Prednisone** (0.5 mg/kg/d for 8-12 weeks,).	Intravenous **methylprednisolone** 1 g/day in 100 mL for three consecutive days followed by oral prednisolone at 0.5 mg/kg per day for 27 days alternating with oral **CTX** at 2 mg/kg for 30 days	Y	Y	FSGS patients were also included in this study, so the baseline of the two groups were unclear
Fu	2012	SC/NB/no	**MMF (**The initial dose was	**CTX** (The dose was 1g once	Y	Y	Langue is

		placebo/RCT	2g/day, then reduced to 1.5g/day after 6 months, 1.0g/day after 18 months, 0.5d/day after 30 months, until the drug is gradually stopped.) **Prednisone** (The initial dose was 1 mg/kg/day once in the morning. After 6-8 weeks, the dose was reduced by 5 mg per week until to 10-20 mg/day.)	a month. After 6 months of continuous use, it was changed to 1g every 3 months, then stopped after 4-6 times of use.) **Prednisone** (The initial dose was 1 mg/kg/day once in the morning. After 6-8 weeks, the dose was reduced by 5 mg per week until to 10-20 mg/day.)			Chinese
Hayati	2019	SC/DB/no placebo/RCT	**MMF** (2 g/d in 2 divided doses for 6 months) **Prednisolone** (0.5 mg/kg/d for 2 to 3 months)	A course of alternate months of steroid in the first, third and fifth months and **CTX** at 1.5 to 2 mg/kg/d in the second, fourth, and sixth months. The steroid months were began with pulse **methylprednisolone**, 1 g intravenously daily for 3 consecutive days, without oral prednisone and then followed by oral prednisolone at 0.5 mg/kg/d for 27 days.	Y	N	The dose of MMF was decreased to 1.5 or 1 g/d in three or two divided doses among patients with gastrointestinal symptoms
**MMF vs Chlorambucil**								
Chan	2007	SC/NB/no placebo/RCT	**MMF** (2.0 g/d in two doses for 6 months) **Prednisolone** was started at 0.8 mg/kg per day p.o., then tapered by 5 mg/day every fortnight until reaching 10 mg/day at around 4 months, then tapered by 2.5 mg/day every fortnight, until total withdrawal at around 6 months from baseline.	**Methylprednisolone** i.v. 1 g daily for 3 days, followed by prednisolone 0.4 mg/kg per day p.o. for 3 weeks, then 0.2 mg/kg per day till the end of the month. Alternating with **chlorambucil** at 0.2 mg/kg per day for 1 month, for a total duration of 6 months	Y	Y	The general characteristics of each group was unclear

**MMF vs CsA**							
Choi	2018	MC/NB/no placebo/RCT	**MMF (**0.5 g twice daily in patients weighing less than 50 kg, or 0.75-1.0g twice daily in patients weighing more than 50 kg) **Prednisolone** (0.15 mg/kg up to a maximum dose of 15 mg/day)	**CsA** (initial dose was 4 mg/kg and the dosage was adjusted to maintain a 100 ± 50 ng/mL trough blood level) **Prednisolone** (0.15 mg/kg up to a maximum dose of 15 mg/day)	Y	Y	None
**CsA vs Steroids**							
Cattran	2001	MC/SB/PC/RCT	**CsA** (initial dose was 3.5 mg/kg and the dosage was adjusted to maintain a 125-225 ug/L trough blood level) **Prednisolone** (0.15 mg/kg up to a maximum dose of 15 mg/day. Dosage was reduced after 26 weeks by thirds at 4-week intervals and was stopped after 8 weeks)	**Placebo** (dosage was 0.035ml /kg/day) **Prednisolone**(0.15 mg/kg up to a maximum dose of 15 mg/day. Dosage was reduced after 26 weeks by thirds at 4-week intervals and was stopped after 8 weeks)	Y	Y	All included patients were steroid-resistant
**CsA vs CTX vs Control**							
Kosmadakis	2010	SC/NB/no placebo/RCT	**Cyclosporine** (3-3.5 mg/kg/day oral) **Methylprednisolone** (12.5 mg/day oral）	**Cyclophosphamide** (2 mg/kg/24 hour oral) **Methylprednisolone** (1.5 mg/kg/48 hour oral)	Y	Y	ACEIs and angiotensin receptor blockers were not prescribed to the other two patients
			Angiotensin-converting enzyme inhibitor (ACEI) **Lisinopril**				
**CsA vs Control**							
Cattran	1995	SC/SB/PC/RCT	**CsA** (initial dose was 3.5 mg/kg and the dosage was adjusted to maintain a 110-170 ug/L trough blood level)	**Placebo** (dosage was 0.035ml /kg/day)	Y	Y	None
**CTX vs Control**							
Donadio	1974	SC/NB/no placebo/RCT	**CTX**(1.5 to 2.5 mg/kg/day by oral administration)	**No drug**	Y	Y	None
Jha	2007	SC/NB/no placebo/RCT	**CTX** (2mg/kg/day by oral administration in the second,	**Supportive therapy** (dietary sodium restriction, diuretics,	Y	Y	ACEI /ARB were

			fourth, and sixth months); Intravenous **methylprednisolone** 1 g/d for 3 consecutive days followed by oral **prednisolone** 0.5mg/kg per d for 27 d in the first, third, and fifth months	and antihypertensive agents)			withheld for at least 1 yr in both groups.
Murphy	1992	SC/NB/no placebo/RCT	**CTX** (1.5mg/kg/day by oral administration for six months)	**Supportive therapy** (dietary sodium restriction, diuretics, and antihypertensive agents)	Y	N	Dipyridamole and warfarin were used in both groups
**CTX vs Chlorambucil**							
Branten	1998	SC/NB/no placebo/RCT	**Methylprednisolone** i.v. 1 g daily for 3 days, followed by prednisolone 0.5 mg/kg per day p.o. for 27 days **Chlorambucil** 0.15 mg/kg per day by oral administration for 1 month, for a total duration of 6 months	**CTX** (1.5-2.0mg/kg/day by oral administration for one year) **Prednisone** 60 mg/day or 125 mg every other day by oral administration for at least 8 weeks	Y	Y	None
Ponticeli	1998	MC/NB/no placebo/RCT	Three cycles of treatment with **methylprednisolone** (1 g given intravenously on 3 consecutive days then 0.4 mg/kg body weight per day given orally for 27 days), each cycle was followed by 1 month of treatment with **CTX**(2.5 mg/kg per day, orally)	Three cycles of treatment with **methylprednisolone** (1 g given intravenously on 3 consecutive days then 0.4 mg/kg body weight per day given orally for 27 days), each cycle was followed by 1 month of treatment with **chlorambucil**(0.2 mg/kg per day, orally)	Y	Y	None
Reichert	1994	SC/NB/no placebo/RCT	Intravenous **CTX** (750 mg/m2 body surface area once every month for 6 months) **Methylprednisolone** (three intravenous 1 g pulses in months 1, 3, and 5).	**Chlorambucil** (0.15 mg/kg body weight per day orally in months 2, 4, and 6) **Prednisone** (three intravenous pulses of 1 g of methylprednisolone followed by oral prednisone at 0.5 mg/kg per day in months 1, 3, and 5)	Y	Y	The patients with serum creatinine levels lower than 150 mmol/L were excluded.

**Steroids vs Control**							
Cameron	1990	SC/SB/PC/RCT	**Prednisone (**125 mg every other day by oral administration for at least 8 weeks, if patients who weighed more than 80 kg, the dosage was 150 mg)	**Placebo**	Y	Y	None
Cattran	1989	SC/NB/no placebo/RCT	**Prednisone (**45 mg per square meter of body-surface area in a single dose on alternate days for six months)	No specific treatment	Y	Y	None
Coggins	1979	SC/DB/PC/RCT	**Prednisone(**Subjects weighing 45 to 80 kg received 125mg, given as a single dose every other morning. Those weighing less received 100mg and those weighing more, 150mg)	**Placebo**	Y	Y	None
**Chlorambucil vs Steroids**							
Ponticeli	1992	MC/NB/no placebo/RCT	Three cycles of treatment with **methylprednisolone** (1 g given intravenously on 3 consecutive days then 0.4mg/kg body weight per day given orally for 27 days), each cycle was followed by 1 month of treatment with **chlorambucil** (0.2 mg/kg per day,orally)	**Methylprednisolone**(1 g given intravenously on 3 consecutive days then 0.4mg/kg body weight per day given orally)	Y	Y	None
**Chlorambucil vs Control**							
Ponticeli	1995	SC/NB/no placebo/RCT	Three cycles of treatment with **methylprednisolone**(1 g given intravenously on 3 consecutive days then 0.4mg/kg body weight per day given orally for 27 days), each cycle was followed by 1 month of treatment with **chlorambucil** (0.2 mg/kg per day,orally)	Symptomatic therapy	Y	Y	None

Ponticeli	1984	SC/NB/no placebo/RCT	Three cycles of treatment with **methylprednisolone** (1 g given intravenously on 3 consecutive days then 0.4mg/kg body weight per day given orally for 27 days), each cycle was followed by 1 month of treatment with **chlorambucil** (0.2 mg/kg per day, orally)	**Supportive therapy** (dietary sodium restriction, diuretics, and antihypertensive agents)	Y	Y	None
Ponticeli	1983	MC/NB/no placebo/RCT	Three cycles of treatment with **methylprednisolone** (1 g given intravenously on 3 consecutive days then 0.4mg/kg body weight per day given orally for 27 days), each cycle was followed by 1 month of treatment with **chlorambucil** (0.2 mg/kg per day, orally)	**Supportive therapy**	Y	N	None
**Rituximab vs Control**							
Dahan	2016	MC/NB/no placebo/RCT	**Rituximab** (Dosage was 375 mg/m^2 by intravenous administration on days 1 and 8 after randomization) **NIAT** (angiotensin–converting enzyme inhibitors and/or angiotensin 2 receptor blockers, diuretics, and statin)	**NIAT** (angiotensin–converting enzyme inhibitors and/or angiotensin 2 receptor blockers, diuretics, and statin)	Y	Y	None
Fervenza	2019	MC/NB/no placebo/RCT	**Rituximab** (Dosage was 100 mg by intravenous administration on days 1 and 15 after randomization)	**CsA** (initial dose was 3.5 mg/kg, divided into two equal doses given at 12-hour intervals. The dosage was adjusted to maintain a 125-175ug/L trough blood level)	Y	Y	None
**Rituximab vs CTX**							
Fernández -Juárez G	2019	MC/NB/no placebo/RCT	Patients received oral **TAC** (0.05 mg/Kg/day), to reach target blood levels of	Patients received **methylprednisolone** at months 1, 3 and 5 (1 g	Y	Y	Treament group is TAC-rituxi

			5–7 ng/mL, for six months. At day 180, patients received intravenous **rituximab** (1 g) and **TAC** dosage was reduced by 25% per month, with complete withdrawal at the end of month 9	intravenously at days 1, 2, 3, then 0.5 mg/Kg/day orally from day 4 to 30). At months 2, 4 and 6, patients received oral **CTX** adjusted for age and renal function (1.0–2.0 mg/Kg/day for 30 days)			mab
Scolari	2021	MC/NB/no placebo/RCT	Patients received **rituximab** at a dose of 1 g on days 1 and 15, without concomitant or subsequent drug therapies. **Methylprednisolone** (2 mg/kg infused in 30 intravenous diluted in 100 mL of normal saline)	The first month of each 2-month cycle (months 1, 3 and 5) beganwith a 1 g pulse of iv. **Methylprednisolone**, repeated daily for three consecutive days followed by oral methylprednisolone (0.4 mg/kg/day) or prednisone (0.5 mg/ kg/day) for the remaining days of that month. In the second month of each 2-month cycle (months 2, 4 and 6), the steroid was stopped and oral **CTX** (2.0 mg/kg/day) was given daily for that month.	Y	Y	None

Note:*MC: Multi centre, SC: Single centre, DB: Double blinded, SB: Single blinded, NB: Non-blinded, PC: Placebo controlled, RCT: Randomized controlled trial.*
