# Supplementary material for: Immunosuppressive treatment for idiopathic membranous nephropathy: An updated network meta-analysis
Source: Open Life Sci. 2023 Jan 10;18(1):20220527. doi: 10.1515/biol-2022-0527 (PMC9835199; doi:10.1515/biol-2022-0527)
Supplement: Supplementary Table 5 [file SupTable_5.Support_for_judgement_about_risk_of_bias.pdf]

Supplementary Table 5. *Support for judgement about risk of bias of included studies*

01. Donadio 1974

Bias	Authors' judgement	Support for judgement
Random sequence generation (selection bias)	Low risk	Randomization was performed by using a table of random numbers.
Allocation concealment (selection bias)	Low risk	The table was maintained by the renal pathologist,and was not seen by the clinicians.
Blinding of participants and personnel (performance bias)	Low risk	Although participants and personnel were not blinded to the remissiong and other outcomes such as 24 hours urinary total protein were unlikely influenced by performance bias
Blinding of outcome assessment (detection bias)	Low risk	remissiong and other outcomes such as 24 hours urinary total protein were unlikely influenced by performance bias
Incomplete outcome data (attrition bias)	Low risk	Analysis was by intention-to-treat principle,details were showed on page 433-436
Selective reporting (reporting bias)	Low risk	Trial protocol was not found but predetermined outcomes in methods section were all reported
Other bias	Low risk	This investigation was supported by a grant from the Mayo Foundation and by Public Health Service grant RR-585 from the National Institutes of Health Clinical Research Center

02. Coggins 1979

Bias	Authors' judgement	Support for judgement
Random sequence generation (selection bias)	Unclear risk	This is a random study,however the detials about randomization are unknow
Allocation concealment (selection bias)	Unclear risk	This information is not clear from the study
Blinding of participants and personnel (performance bias)	Low risk	This is a double-blind study, remission and other outcomes were unlikely influenced
Blinding of outcome assessment (detection bias)	Low risk	Remission and other outcomes were not affected since this is a double-blind study
Incomplete outcome data (attrition bias)	Low risk	Analysis was by intention-to-treat principle,details were showed on page 1303-1305
Selective reporting (reporting bias)	Low risk	Trial protocol was not found but predetermined outcomes in methods section were all reported
Other bias	Unclear risk	This information is not clear from the study

03. Ponticelli 1983

Bias	Authors' judgement	Support for judgement
Random sequence generation (selection bias)	Low risk	patients with membranous nephropathy and nephrotic syndrome were admitted to the study and then they were randomly allocated treatment or not through a sealed envelope system using a single randomisation list in our centre.
Allocation concealment (selection bias)	Low risk	patients with membranous nephropathy and nephrotic syndrome were admitted to the study and then they were randomly allocated treatment or not through a sealed envelope system using a single randomisation list in our centre.

Blinding of participants and personnel (performance bias)	Low risk	Although participants and personnel were not blinded to the intervention received, remission was unlikely influenced by performance bias
Blinding of outcome assessment (detection bias)	Low risk	Remission was not affected by detection bias
Incomplete outcome data (attrition bias)	Low risk	Analysis was by intention-to-treat principle,details were showed
Selective reporting (reporting bias)	Low risk	Trial protocol was not found but predetermined outcomes in methods section were all reported
Other bias	Unclear risk	This information is not clear from the study

04. Ponticelli 1984

Bias	Authors' judgement	Support for judgement
Random sequence generation (selection bias)	Low risk	The patients were randomly assigned from a table of random numbers.
Allocation concealment (selection bias)	Unclear risk	This information is not clear from the study
Blinding of participants and personnel (performance bias)	Low risk	Although participants and personnel were not blinded to the intervention received, remission was unlikely influenced by performance bias
Blinding of outcome assessment (detection bias)	Low risk	Remission was not affected by detection bias
Incomplete outcome data (attrition bias)	Low risk	Analysis was by intention-to-treat principle,details were showed on page 947-948
Selective reporting (reporting bias)	Low risk	Trial protocol was not found but predetermined outcomes in methods section were all reported
Other bias	Unclear risk	This information is not clear from the study

05. Cattran 1989

Bias	Authors' judgement	Support for judgement
Random sequence generation (selection bias)	Low risk	The patients were assigned by the trial coordinator according to a table of random numbers.
Allocation concealment (selection bias)	Low risk	Treatment allocation was concealed to investigators by "computer"
Blinding of participants and personnel (performance bias)	Low risk	Although participants and personnel were not blinded to the intervention received, remission and renal survival outcomes were unlikely influenced by performance bias
Blinding of outcome assessment (detection bias)	Low risk	Remission and renal survival outcomes were not affected by detection bias
Incomplete outcome data (attrition bias)	Low risk	Analysis was by intention-to-treat principle,details were showed on page 211-214
Selective reporting (reporting bias)	Low risk	Trial protocol was not found but predetermined outcomes in methods section were all reported
Other bias	Unclear risk	This information is not clear from the study

06.Cameron 1990

Bias	Authors' judgement	Support for judgement
Random sequence generation (selection bias)	Low risk	Randomization was performed centrally, and coded tablets given locallyfrom bottles supplied from the co-ordinator
Allocation concealment (selection bias)	Low risk	Treatment allocation was concealed to investigators by "central random"
Blinding of participants and personnel (performance bias)	Low risk	Although participants and personnel were not blinded to the intervention received, remission and other outcomes such as renal function were unlikely influenced by performance bias
Blinding of outcome assessment (detection bias)	Low risk	Remission and other outcomes such as renal function were not affected by detection bias
Incomplete outcome data (attrition bias)	Low risk	Analysis was by intention-to-treat principle,details were showed on page 135-146
Selective reporting (reporting bias)	Low risk	Trial protocol was not found but predetermined outcomes in methods section were all reported
Other bias	Unclear risk	This information is not clear from the study

07.Murphy 1992

Bias	Authors' judgement	Support for judgement
Random sequence generation (selection bias)	Low risk	After consent was obtained,randomization was performed by opening sealed envelopes.
Allocation concealment (selection bias)	Low risk	Treatment allocation was concealed to investigators by "sealed envelope"
Blinding of participants and personnel (performance bias)	Low risk	Although participants and personnel were not blinded to the intervention received, remission and other outcomes such as renal function were unlikely influenced by performance bias
Blinding of outcome assessment (detection bias)	Low risk	remission and other outcomes were not affected by detection bias
Incomplete outcome data (attrition bias)	High risk	The details for adverse events were not reported

Selective reporting (reporting bias)	High risk	The details for adverse events were not reported
Other bias	Unclear risk	This information is not clear from the study

08.Ponticelli 1992

Bias	Authors' judgement	Support for judgement
Random sequence generation (selection bias)	Low risk	The coordinatiing center assigned the patients consecutively to one of the two treament regiments in random order.
Allocation concealment (selection bias)	Low risk	Treatment allocation was concealed to investigators by "center random"
Blinding of participants and personnel (performance bias)	Low risk	Although participants and personnel were not blinded to the intervention received, remission and other outcomes such as 24 hours urinary total protein were unlikely influenced by performance bias
Blinding of outcome assessment (detection bias)	Low risk	remission and other outcomes such as 24 hours urinary total protein were not affected by detection bias
Incomplete outcome data (attrition bias)	Low risk	Analysis was by intention-to-treat principle,details were showed on page 600-602
Selective reporting (reporting bias)	Low risk	Trial protocol was not found but predetermined outcomes in methods section were all reported
Other bias	Unclear risk	This information is not clear from the study

09.Reichert 1994

Bias	Authors' judgement	Support for judgement
Random sequence generation (selection bias)	Unclear risk	This is a random study,however there is no detial about the randomization.
Allocation concealment (selection bias)	Unclear risk	This information is not clear from the study
Blinding of participants and personnel (performance bias)	Low risk	Although participants and personnel were not blinded to the intervention received, remission was unlikely influenced by performance bias
Blinding of outcome assessment (detection bias)	Low risk	Remission was not affected by detection bias
Incomplete outcome data (attrition bias)	Low risk	Analysis was by intention-to-treat principle,details were showed on page 328-331
Selective reporting (reporting bias)	Low risk	Trial protocol was not found but predetermined outcomes in methods section were all reported
Other bias	Low risk	This work was supported by NWO grant 900/716-111 from the Netherlands Foundation of Scientific Research.

10. Cattran 1995

Bias	Authors' judgement	Support for judgement
Random sequence generation (selection bias)	Unclear risk	This is a random study,however there is no detial about the randomization.
Allocation concealment (selection bias)	Unclear risk	This information is not clear from the study
Blinding of participants and personnel (performance bias)	Low risk	This study is single-blind for patients,remission and other outcomes such as renal function were unlikely influenced by performance bias

Blinding of outcome assessment (detection bias)	Low risk	Remission and other outcomes such as renal function were were not affected by detection bias
Incomplete outcome data (attrition bias)	Low risk	Analysis was by intention-to-treat principle,details were showed on page 1131-1132
Selective reporting (reporting bias)	Low risk	Trial protocol was not found but predetermined outcomes in methods section were all reported
Other bias	Unclear risk	This information is not clear from the study

11.Ponticelli 1995

**Bias**	**Authors' judgement**	**Support for judgement**
Random sequence generation (selection bias)	Unclear risk	This is a random study,however there is no detial about the randomization.
Allocation concealment (selection bias)	Unclear risk	This information is not clear from the study
Blinding of participants and personnel (performance bias)	Low risk	Although participants and personnel were not blinded to the intervention received, remission was unlikely influenced by performance bias
Blinding of outcome assessment (detection bias)	Low risk	Remission was not affected by detection bias
Incomplete outcome data (attrition bias)	High risk	The predetermined outcomes was not shown,we suspect the completeness of the outcome
Selective reporting (reporting bias)	High risk	Trial protocol was not found,we suspect that the outcomes of this study was reported selectively
Other bias	Unclear risk	This information is not clear from the study

12.Branten 1998

Bias	Authors' judgement	Support for judgement
Random sequence generation (selection bias)	Unclear risk	This is a random study,however there is no detial about the randomization.
Allocation concealment (selection bias)	Unclear risk	This information is not clear from the study
Blinding of participants and personnel (performance bias)	Low risk	Although participants and personnel were not blinded to the intervention received, remission and other outcomes were unlikely influenced by performance bias
Blinding of outcome assessment (detection bias)	Low risk	Remission and other outcomes were not affected by detection bias
Incomplete outcome data (attrition bias)	Low risk	Analysis was by intention-to-treat principle,details were showed on page 361-365
Selective reporting (reporting bias)	Low risk	Trial protocol was not found but predetermined outcomes in methods section were all reported
Other bias	Unclear risk	This information is not clear from the study

13.Ponticelli 1998

Bias	Authors' judgement	Support for judgement
Random sequence generation (selection bias)	Low risk	Patients were assigned consecutively to one of the two treatruent regimens, according to a center-stratified random order
Allocation concealment (selection bias)	Low risk	Treatment allocation was concealed to investigators by "center random"
Blinding of participants and personnel (performance bias)	Low risk	Although participants and personnel were not blinded to the intervention received, remission and other outcomes such as 24 hours urinary total protein were unlikely influenced by performance bias
Blinding of outcome assessment (detection bias)	Low risk	remission and other outcomes such as 24 hours urinary total protein were not affected by detection bias
Incomplete outcome data (attrition bias)	Unclear risk	We suspect the completeness of the adverse events
Selective reporting (reporting bias)	Unclear risk	Trial protocol was not found and the adverse events were not complete.
Other bias	Low risk	This work was supported in part by a grant from Ospedabc Maggiore di Milano.

14.Cattran 2001

Bias	Authors' judgement	Support for judgement
Random sequence generation (selection bias)	Low risk	Randomization was performed by the clinical coordinating center from a table of random numbers and was stratified by center in blocks of two to ensure a balance between groups.
Allocation concealment (selection bias)	Low risk	Treatment allocation was concealed to investigators by "computer"
Blinding of participants and personnel (performance bias)	Low risk	This study is single-blind for patients,remission and other outcomes such as renal function were unlikely influenced by performance bias

Blinding of outcome assessment (detection bias)	Low risk	Remission and other outcomes such as renal function were were not affected by detection bias
Incomplete outcome data (attrition bias)	Low risk	Analysis was by intention-to-treat principle,details were showed on page 1486-1488
Selective reporting (reporting bias)	Low risk	Trial protocol was not found but predetermined outcomes in methods section were all reported
Other bias	Low risk	The study was supported by the Kidney Foundation of Canada and Novartis Canada

15.Jha 2007

Bias	Authors' judgement	Support for judgement
Random sequence generation (selection bias)	Low risk	Patients were divided into two groups using table of random numbers.
Allocation concealment (selection bias)	Unclear risk	This information is not clear from the study
Blinding of participants and personnel (performance bias)	Low risk	Although participants and personnel were not blinded to the remissiong and other outcomes such as renal function were unlikely influenced by performance bias
Blinding of outcome assessment (detection bias)	Low risk	remissiong and other outcomes such as renal function were not affected by detection bias
Incomplete outcome data (attrition bias)	Low risk	Analysis was by intention-to-treat principle,details were showed on page 1900-1902
Selective reporting (reporting bias)	Low risk	Trial protocol was not found but predetermined outcomes in methods section were all reported
Other bias	Unclear risk	This information is not clear from the study

16.Praga2007

Bias	Authors' judgement	Support for judgement
Random sequence generation (selection bias)	Low risk	Randomization was performed by the clinical coordinating center using a table of random numbers and was stratified by centers
Allocation concealment (selection bias)	Low risk	Allocation concealment was performed by enclosing assignments in sequentially numbered, opaque-closed envelopes.
Blinding of participants and personnel (performance bias)	Low risk	Although participants and personnel were not blinded to the intervention received, remission and renal survival outcomes were unlikely influenced by performance bias
Blinding of outcome assessment (detection bias)	Low risk	Remission and renal survival outcomes were not affected by detection bias
Incomplete outcome data (attrition bias)	Low risk	Analysis was by intention-to-treat principle,details were showed on page 925-927
Selective reporting (reporting bias)	Low risk	This clinical trial is registered in The Cochrane Renal Group Registry(CRG100500015).
Other bias	Unclear risk	This information is not clear from the study

17.chan2007

Bias	Authors' judgement	Support for judgement
Random sequence generation (selection bias)	Low risk	Patients who satisfied the selection criteria were randomized by drawing envelope into either one of two treatment groups
Allocation concealment (selection bias)	Low risk	Treatment allocation was concealed to investigators by "drawing envelope"
Blinding of participants and personnel (performance bias)	Low risk	Although participants and personnel were not blinded to the intervention received, remission and other outcomes were unlikely influenced by performance bias
Blinding of outcome assessment (detection bias)	Low risk	Remission and other outcomes were not affected by detection bias
Incomplete outcome data (attrition bias)	Low risk	Analysis was by intention-to-treat principle,details were showed on page 577-580
Selective reporting (reporting bias)	Low risk	Trial protocol was not found but predetermined outcomes in methods section were all reported
Other bias	Unclear risk	This information is not clear from the study

18. Dussol 2008

Bias	Authors' judgement	Support for judgement
Random sequence generation (selection bias)	Low risk	Randomization was performed by each center through a centralized Internet on-line application provided by the sponsor (minimization method)
Allocation concealment (selection bias)	Low risk	Treatment allocation was concealed to investigators by "computer"
Blinding of participants and personnel (performance bias)	Low risk	Although participants and personnel were not blinded to the intervention received, remission and renal survival outcomes were unlikely influenced by performance bias

Blinding of outcome assessment (detection bias)	Low risk	Remission and renal survival outcomes were not affected by detection bias
Incomplete outcome data (attrition bias)	Low risk	Analysis was by intention-to-treat principle,details were showed on page 700-702
Selective reporting (reporting bias)	Low risk	Trial protocol was published (CRG020800132). Reported protocol’s predetermined outcomes
Other bias	Unclear risk	This information is not clear from the study

19.Senthil 2008

Bias	Authors' judgement	Support for judgement
Random sequence generation (selection bias)	Low risk	Treatment allocation was on the basis of minimization.Minimization is a valid alternative to randomization, and ensures uniformity between the two groups with respect to the characteristics used in the allocation process.
Allocation concealment (selection bias)	Unclear risk	This information is not clear from the study
Blinding of participants and personnel (performance bias)	Low risk	Although participants and personnel were not blinded to the intervention received, remission and mean proteinuria over creatinuria ratio were unlikely influenced by performance bias
Blinding of outcome assessment (detection bias)	Low risk	Remission and mean proteinuria over creatinuria ratio were not affected by detection bias
Incomplete outcome data (attrition bias)	High risk	FSGS patients were also included in this study,so the baseline and some results of the two groups were unclear
Selective reporting (reporting bias)	Unclear risk	Trial protocol was not found and this information is not clear from the study
Other bias	Unclear risk	This information is not clear from the study

20.Chen 2010

Bias	Authors' judgement	Support for judgement
Random sequence generation (selection bias)	Low risk	Randomization was performed by a clinical coordinating center using a table of random numbers and was stratified by centers.
Allocation concealment (selection bias)	Low risk	Allocation concealment was performed by enclosing assignments in sequentially numbered, opaque-closed envelopes
Blinding of participants and personnel (performance bias)	Low risk	Although participants and personnel were not blinded to the intervention received, remission and renal survival outcomes were unlikely influenced by performance bias
Blinding of outcome assessment (detection bias)	Low risk	Remission and renal survival outcomes were not affected by detection bias
Incomplete outcome data (attrition bias)	Low risk	Analysis was by intention-to-treat principle,details were showed on page 235-236
Selective reporting (reporting bias)	Low risk	Trial protocol was not found but predetermined outcomes in methods section were all reported
Other bias	Unclear risk	This information is not clear from the study

21.Kosmadakis2010

Bias	Authors' judgement	Support for judgement
Random sequence generation (selection bias)	Unclear risk	0This is a random study,however the detials about the randomization were not mentioned
Allocation concealment (selection bias)	Low risk	The person doing the randomization was blinded, that is, did not have the right to recruit and did not have direct contact with any of the patients.
Blinding of participants and personnel (performance bias)	Low risk	Although participants and personnel were not blinded to the intervention received, remission and other outcomes were unlikely influenced by performance bias
Blinding of outcome assessment (detection bias)	Low risk	Remission and renal survival outcomes were not affected by detection bias
Incomplete outcome data (attrition bias)	Low risk	Analysis was by intention-to-treat principle,details were showed .
Selective reporting (reporting bias)	Low risk	Trial protocol was not found but predetermined outcomes in methods section were all reported
Other bias	Low risk	the investigators had no external funding for this study and no reason to be biased for or against a specific substance.

22.Fu 2012

Bias	Authors' judgement	Support for judgement
Random sequence generation (selection bias)	Unclear risk	This is a random study,however the detials about the randomization were not mentioned
Allocation concealment (selection bias)	Unclear risk	This information is not clear from the study

Blinding of participants and personnel (performance bias)	Low risk	Although participants and personnel were not blinded to the remissiong and other outcomes such as renal function were unlikely influenced by performance bias
Blinding of outcome assessment (detection bias)	Low risk	remission and other outcomes were not affected by detection bias
Incomplete outcome data (attrition bias)	Low risk	Analysis was by intention-to-treat principle,details were showed
Selective reporting (reporting bias)	Low risk	Trial protocol was not found but predetermined outcomes in methods section were all reported
Other bias	Unclear risk	This information is not clear from the study

23.Xu 2013

Bias	Authors' judgement	Support for judgement
Random sequence generation (selection bias)	Unclear risk	This is a random study,however the detials about the randomization were not mentioned
Allocation concealment (selection bias)	Unclear risk	This information is not clear from the study
Blinding of participants and personnel (performance bias)	Low risk	Although participants and personnel were not blinded to the intervention received, remission and renal survival outcomes were unlikely influenced by performance bias
Blinding of outcome assessment (detection bias)	Low risk	Remission and renal survival outcomes were not affected by detection bias
Incomplete outcome data (attrition bias)	Low risk	Analysis was by intention-to-treat principle,details were showed on page 155-159
Selective reporting (reporting bias)	Low risk	Trial protocol was not found but predetermined outcomes in methods section were all reported
Other bias	Low risk	This work was supported by grants from the National Basic Research Program of China 973, grant No. 2012CB517600 (grant No. 2012CB517604), the Research on Hypertensive Nephropathy and Ischemic Kidney Diseases National Key Technology R&D Program (12-5), grant No. 2011BAI10B00

		(grant No. 2011BAI10B06), and the National Natural Science Foundation of China (grant No. 30871001).

24.He 2013

Bias	Authors' judgement	Support for judgement
Random sequence generation (selection bias)	Low risk	The randomization was performed through a preprinted randomization table.
Allocation concealment (selection bias)	Unclear risk	This information is not clear from the study
Blinding of participants and personnel (performance bias)	Low risk	Although participants and personnel were not blinded to the intervention received, remission and renal survival outcomes were unlikely influenced by performance bias
Blinding of outcome assessment (detection bias)	Low risk	Remission and renal survival outcomes were not affected by detection bias
Incomplete outcome data (attrition bias)	Low risk	Analysis was by intention-to-treat principle,details were showed on page 567-568
Selective reporting (reporting bias)	Low risk	Trial protocol was not found but predetermined outcomes in methods section were all reported
Other bias	Unclear risk	This information is not clear from the study

25.Dahan2016

Bias	Authors' judgement	Support for judgement
Random sequence generation (selection bias)	Unclear risk	This is a random study,however there is no detial about the randomization.
Allocation concealment (selection bias)	Unclear risk	This information is not clear from the study
Blinding of participants and personnel (performance bias)	Low risk	Although participants and personnel were not blinded to the intervention received, remission and other outcomes such as PLA2R were unlikely influenced by performance bias
Blinding of outcome assessment (detection bias)	Low risk	remission and other outcomes such as PLA2R were not affected by detection bias
Incomplete outcome data (attrition bias)	Low risk	Analysis was by intention-to-treat principle,details were showed on page 2-5
Selective reporting (reporting bias)	Low risk	Trial protocol was published (NCT01508468)). Reported protocol' s predetermined outcomes.
Other bias	Low risk	This study was funded by Programme Hospitalier de Recherche Clinique, French Ministry of Health grant AOM10089; European Research Council ERC-2012-ADG_20120314 grant agreement 322947; Agence Nationale pour la Recherche Programme Blanc SVSE1 (2012) Decision grant ANR-12-BSE1-0002-01; Fondation pour la Recherche Médicale Equipe FRM 2012 grant; and 7th Framework Programme of the European Community contract 2012-305608 (European Consortium for High-Throughput Research in Rare Kidney Diseases).

26.Peng 2016

Bias	Authors' judgement	Support for judgement
Random sequence generation (selection bias)	Unclear risk	This is a random study,however there is no detial about the randomization.

Allocation concealment (selection bias)	Unclear risk	This information is not clear from the study
Blinding of participants and personnel (performance bias)	Low risk	Although participants and personnel were not blinded to the intervention received, remission was unlikely influenced by performance bias
Blinding of outcome assessment (detection bias)	Low risk	Remission was not affected by detection bias
Incomplete outcome data (attrition bias)	Low risk	Analysis was by intention-to-treat principle,details were showed on page 3-5
Selective reporting (reporting bias)	Low risk	Trial protocol was not found but predetermined outcomes in methods section were all reported
Other bias	Low risk	This study was supported by research grants as follows: 1)National Basic Research Program of China 973 Program (No.2012CB517602); 2) National Natural Science Foundation of China (81370812); 3) Doctoral Program of Ministry of Education of China (20122307110018); 4) Special Grade of China Postdoctoral Science Foundation (No. 201003463); 5)Heilongjiang Postdoctoral Science Research Foundation(No. LBHQ10028).

27.Li 2017

Bias	Authors' judgement	Support for judgement
Random sequence generation (selection bias)	Low risk	Patients were randomized into two groups according to a randomization list generated from the table of random numbers.
Allocation concealment (selection bias)	Unclear risk	This information is not clear from the study
Blinding of participants and personnel (performance bias)	Low risk	Although participants and personnel were not blinded to the remissiong and other outcomes such as renal function were unlikely influenced by performance bias
Blinding of outcome assessment (detection bias)	Low risk	Remissiong and other outcomes such as renal function were not affected by detection bias
Incomplete outcome data (attrition bias)	Low risk	Analysis was by intention-to-treat principle,details were showed on page 9766-9767
Selective reporting (reporting bias)	Low risk	Trial protocol was not found but predetermined outcomes in methods section were all reported
Other bias	Low risk	This study was supported by research grants as follows: 1) National Natural Science Foundation of China (81300605); 2) Major Medical Science and Technology Program Plan of Henan Province (201501010)

28. Ramachandran 2017

Bias	Authors' judgement	Support for judgement
Random sequence generation (selection bias)	Low risk	Participants were randomly assigned following computer based random numbers to one of the two treatmentgroups.
Allocation concealment (selection bias)	Low risk	Treatment allocation was concealed to investigators by "sequentially labelled sealed envelopes"
Blinding of participants and personnel (performance bias)	Low risk	Although participants and personnel were not blinded to the intervention received, remission and reGFR and adverse events were unlikely influenced by performance bias
Blinding of outcome assessment (detection bias)	Low risk	Remission and eGFR and adverse events were not affected by detection bias
Incomplete outcome data (attrition bias)	Low risk	Analysis was by intention-to-treat principle,details were showed on page 140-142
Selective reporting (reporting bias)	Low risk	Trial protocol was not found but predetermined outcomes in methods section were all reported
Other bias	Low risk	The first author received scientific grant from the Indian Society of Nephrology.

29.Liang2017

Bias	Authors' judgement	Support for judgement
Random sequence generation (selection bias)	High risk	Most of the patients were randomly assigned, whereas a few patients had a strong desire to choose the regimen.
Allocation concealment (selection bias)	High risk	A few patients chosed the regimen independently,it can influence the baseline of the patients in two groups.
Blinding of participants and personnel (performance bias)	Low risk	Although participants and personnel were not blinded to the intervention received, remission and other outcomes were unlikely influenced by performance bias
Blinding of outcome assessment (detection bias)	Low risk	Remission and other outcomes were not affected by detection bias
Incomplete outcome data (attrition bias)	Low risk	Analysis was by intention-to-treat principle,details were showed on page 514-516
Selective reporting (reporting bias)	Low risk	Trial protocol was not found but predetermined outcomes in methods section were all reported
Other bias	Unclear risk	This information is not clear from the study

30.Choi 2018

Bias	Authors' judgement	Support for judgement
Random sequence generation (selection bias)	Low risk	All patients were assigned using a block randomization technique,The table of random numbers was generated using the SAS randomization program
Allocation concealment (selection bias)	Low risk	Allocation concealment was done by sealed sequentially numbered opaque envelopes.
Blinding of participants and personnel (performance bias)	Low risk	Although participants and personnel were not blinded to the intervention received, remission and other outcomes were unlikely influenced by performance bias

Blinding of outcome assessment (detection bias)	Low risk	Remission and other outcomes were not affected by detection bias
Incomplete outcome data (attrition bias)	Low risk	Analysis was by intention-to-treat principle,details were showed on page 5-9
Selective reporting (reporting bias)	Low risk	Trial protocol was published (NCT01282073). Reported protocol’s predetermined outcomes
Other bias	Low risk	This work was supported by a grant from the Korea Health Technology R & D Project through the Korea Health Industry Development Institute (KHIDI), funded by the Ministry of Health & Welfare, Republic of Korea (grant number: HC15C1129; HI15C0001; HI13C1232).

31.Hayati2019

Bias	Authors' judgement	Support for judgement
Random sequence generation (selection bias)	Low risk	Patients were randomly divided into two groups by randomized blocks
Allocation concealment (selection bias)	Unclear risk	This information is not clear from the study
Blinding of participants and personnel (performance bias)	Low risk	Single blind. the person who evaluated patient don't know the kind of drug
Blinding of outcome assessment (detection bias)	Low risk	remission and other outcomes were not affected by detection bias
Incomplete outcome data (attrition bias)	Low risk	Analysis was by intention-to-treat principle,details were showed
Selective reporting (reporting bias)	Low risk	Trial protocol was published (available at https://irct.ir/trial/29433). Reported protocol’s predetermined outcomes
Other bias	Low risk	The study was a double-blind, randomized and controlled clinical trial approved by the Research Center of Ahvaz Jundishapur University of Medical Sciences. Our study performed at the outpatient clinic of nephrology in the

		Golestan hospital, Ahvaz, Iran. The drugs (MMF and cyclophosphamide) were provided to the patients free of cost.

32.Fervenza2019

Bias	Authors' judgement	Support for judgement
Random sequence generation (selection bias)	Low risk	The random-<br>ization schedule was computer-generated
Allocation concealment (selection bias)	Low risk	The random-<br>ization schedule was computer-generated, strati-<br>fied according to site, blocked with randomly<br>varied block sizes of two and four, and con-<br>cealed with the use of a Web-based, locked cen-<br>tral randomization system.
Blinding of participants and personnel (performance bias)	Low risk	outcomes were unlikely influenced by performance bias
Blinding of outcome assessment (detection bias)	Low risk	remission and other outcomes were not affected by detection bias
Incomplete outcome data (attrition bias)	Low risk	Analysis was by intention-to-treat principle,details were showed
Selective reporting (reporting bias)	Low risk	Trial protocol was published (available at NEJM.org). Reported protocol's predetermined outcomes
Other bias	Low risk	The funders had no<br>role in the trial design or conduct; the collection,<br>management, analysis, or interpretation of the<br>data; or in the preparation or review of the manu-<br>script or the approval of the manuscript for sub-<br>mission.

33.Scolari2021

Bias	Authors' judgement	Support for judgement
Random sequence generation (selection bias)	Low risk	Participants were randomized 1:1 to the intervention or active comparator arm.
Allocation concealment (selection bias)	Low risk	An analyst from a distant site, with no clin-<br>ical involvement in the trial, generated the randomization lists and kept them concealed.
Blinding of participants and personnel (performance bias)	Low risk	Although participants and personnel were not blinded to the intervention received, remission and other outcomes were unlikely influenced by performance bias
Blinding of outcome assessment (detection bias)	Low risk	remission and other outcomes were not affected by detection bias
Incomplete outcome data (attrition bias)	Low risk	Analysis was by intention-to-treat principle,details were showed
Selective reporting (reporting bias)	Low risk	NCT03018535
Other bias	Low risk	The interpretation and conclusions contained herein are those of the re-<br>searchers. All authors affirm that this manuscript is an honest, accurate, and<br>transparent account of the study being reported; that no important aspects of<br>the study have been omitted; and that any discrepancies from the study as<br>planned have been explained.

34.Fernández-Juárez G.2021

Bias	Authors' judgement	Support for judgement
Random sequence generation (selection bias)	Low risk	We used a random number producing algorithm by central computer systems for simple<br>randomization, with an equal allocation ratio (1:1) to intervention with corticosteroid-<br>cyclophosphamide or tacrolimus-rituximab.
Allocation concealment (selection bias)	Low risk	The subject numbers were assigned<br>sequentially as each subject entered the study.
Blinding of participants and personnel (performance bias)	Low risk	Although participants and personnel were not blinded to the intervention received, remission and other outcomes were unlikely influenced by performance bias

Blinding of outcome assessment (detection bias)	Low risk	remission and other outcomes were not affected by detection bias
Incomplete outcome data (attrition bias)	Low risk	Analysis was by intention-to-treat principle,details were showed
Selective reporting (reporting bias)	Low risk	The trial is<br>registered at ClinicalTrials.gov NCT01955187
Other bias	Low risk	Acknowledgements. Work in this study was supported by the Instituto de Salud Carlos<br>III / Fondo Europeo de Desarrollo Regional (ISCIII/FEDER) grants PI13/02495 and<br>ICI14/00350, Red de Investigación Renal (RedInRen) (RD12/0021/0029), European<br>Renal Association-European Dialysis and Transplant Association (ERA-EDTA),<br>Fundación Renal Iñigo Álvarez de Toledo (FRIAT), and Fundación para la<br>Investigación Biomédica Hospital 12 de Octubre (i+12). Funding agencies had no<br>participation in any of the phases of the study.<br>We thank the trial staff at all participating sites and the patients who participated in the<br>trial.

Footnotes
