# Supplementary material for: Immunosuppressive treatment for idiopathic membranous nephropathy: An updated network meta-analysis
Source: Open Life Sci. 2023 Jan 10;18(1):20220527. doi: 10.1515/biol-2022-0527 (PMC9835199; doi:10.1515/biol-2022-0527)
Supplement: Supplementary Table 6 [file SupTable_6.Details_of_GRADE_assessment_for_CR.pdf]

Supplementary Table 6. *Details of GRADE assessment for CR*

Quality assessment							No of patients		Effect		Quality	Importance
**No of studies**	**Design**	**Risk of bias**	**Inconsistency**	**Indirectness**	**Imprecision**	**Other considerations**	**CR**	**Control**	**Relative (95% CI)**	**Absolute**		
**New Outcome**												
31	no methodology chosen					none	271/965 (28.1%)	217/944 (23%)	OR 1.33 (1.08 to 1.63)	54 more per 1000 (from 14 more to 97 more)		
								20.1%		50 more per 1000 (from 13 more to 90 more)		
**New Outcome - TAC VS CTX (follow-up mean 14.5 months)**												
6	randomised trials	no serious risk of bias[1]	serious[2]	no serious indirectness	serious[3]	reporting bias[4]	84/210 (40%)	87/207 (42%)	OR 0.93 (0.64 to 1.37)	18 fewer per 1000 (from 103 fewer to 78 more)	⊕OOO VERY LOW	
								35.7%		16 fewer per 1000 (from 95 fewer to 75 more)		
**New Outcome - TAC VS CON (follow-up mean 30 months)**												
1	randomised trials	no serious risk of bias	no serious inconsistency	no serious indirectness	serious[5]	reporting bias[6]	3/25 (12%)	5/23 (21.7%)	OR 0.49 (0.1 to 2.34)	98 fewer per 1000 (from 190 fewer to 177 more)	⊕⊕OO LOW	
								21.7%		97 fewer per 1000 (from 190 fewer to 176 more)		
**New Outcome - MMF VS CON (follow-up mean 12 months)**												
1	randomised trials	no serious risk of bias	no serious inconsistency	no serious indirectness	serious[7]	reporting bias[8]	1/19 (5.3%)	2/17 (11.8%)	OR 0.42 (0.03 to 5.06)	65 fewer per 1000 (from 114 fewer to 285 more)	⊕⊕OO LOW	
								11.8%		65 fewer per 1000 (from 114 fewer to 286 more)		
**New Outcome - MMF VS CTX (follow-up mean 15 75 months)**												
4	randomised trials	no serious risk of bias	no serious inconsistency[9]	no serious indirectness	serious[10]	none[11]	18/71 (25.4%)	19/66 (28.8%)	OR 0.87 (0.4 to 1.9)	28 fewer per 1000 (from 149 fewer to 147 more)	⊕⊕⊕O MODERATE	
								28.3%		27 fewer per 1000 (from 147 fewer to 146 more)		
**New Outcome - MMF VS CH (follow-up mean 15 months)**												
1	randomised trials	no serious risk of bias	no serious inconsistency	no serious indirectness	serious[12]	reporting bias[13]	2/11 (18.2%)	3/9 (33.3%)	OR 0.44 (0.06 to 3.51)	153 fewer per 1000 (from 304 fewer to 304 more)	⊕⊕OO LOW	
								33.3%		153 fewer per 1000 (from 304 fewer to 304 more)		
**New Outcome - MMF vs CSA (follow-up mean 11 2 months)**												
1	randomised trials	no serious risk of	no serious inconsistency	no serious indirectness	serious[14]	reporting bias[15]	4/21 (19%)	3/18 (16.7%)	OR 1.18 (0.23 to 6.13)	24 more per 1000 (from 123	⊕⊕OO LOW	

		bias								fewer to 384 more)		
								16.7%		24 more per 1000 (from 123 fewer to 384 more)		
**New Outcome - CSA VS STE (follow-up mean 18 months)**												
1	randomised trials	no serious risk of bias	no serious inconsistency	no serious indirectness	serious[16]	reporting bias[17]	2/28 (7.1%)	1/23 (4.3%)	OR 1.69 (0.14 to 19.94)	28 more per 1000 (from 37 fewer to 432 more)	⊕⊕OO LOW	
								4.4%		28 more per 1000 (from 38 fewer to 435 more)		
**New Outcome - CTX VS CON (follow-up mean 52 months)**												
3	randomised trials	no serious risk of bias	no serious inconsistency[18]	no serious indirectness	serious[19]	reporting bias[20]	6/38 (15.8%)	1/42 (2.4%)	OR 6.26 (1.02 to 38.45)	109 more per 1000 (from 0 more to 460 more)	⊕⊕OO LOW	
								0%		-		
**New Outcome - CTX VS CH (follow-up mean 22 75 months)**												
3	randomised trials	no serious risk of bias	no serious inconsistency[21]	no serious indirectness	serious[22]	none[23]	23/69 (33.3%)	13/68 (19.1%)	OR 2.14 (0.98 to 4.7)	145 more per 1000 (from 3 fewer to 335 more)	⊕⊕⊕O MODERATE	
								11.1%		100 more per 1000 (from 2 fewer to 259 more)		
**New Outcome - STE vs CON (follow-up mean 35.5 months)**												
2	randomised trials	no serious risk of bias	no serious inconsistency[24]	no serious indirectness	serious[25]	reporting bias[26]	20/115 (17.4%)	23/115 (20%)	OR 0.82 (0.42 to 1.6)	30 fewer per 1000 (from 105 fewer to 86 more)	⊕⊕OO LOW	
								17.6%		27 fewer per 1000 (from 94 fewer to 79 more)		
**New Outcome - CH vs STE (follow-up mean 48 months)**												
1	randomised trials	no serious risk of bias	no serious inconsistency	no serious indirectness	serious[27]	reporting bias[28]	14/45 (31.1%)	10/47 (21.3%)	OR 1.67 (0.65 to 4.28)	98 more per 1000 (from 63 fewer to 324 more)	⊕⊕OO LOW	
								21.3%		98 more per 1000 (from 63 fewer to 324 more)		
**New Outcome - CH vs CON (follow-up mean 49.6 months)**												
3	randomised trials	no serious risk of bias	no serious inconsistency[29]	no serious indirectness	no serious imprecision[30]	none[31]	38/95 (40%)	7/93 (7.5%)	OR 8.43 (3.49 to 20.38)	332 more per 1000 (from 146 more to 549 more)	⊕⊕⊕⊕ HIGH	
								6.7%		310 more per 1000 (from 133 more to 527 more)		
**New Outcome - RTX vs CON (follow-up mean 17 months)**												
1	randomised trials	no serious risk of bias	no serious inconsistency	no serious indirectness	serious[32]	reporting bias[33]	7/37 (18.9%)	1/38 (2.6%)	OR 8.63 (1.01 to 74.11)	163 more per 1000 (from 0 more to 641 more)	⊕⊕OO LOW	

								2.6%		161 more per 1000 (from 0 more to 638 more)		
**New Outcome - TAC VS CSA (follow-up mean 6 months)**												
1	randomised trials	no serious risk of bias	no serious inconsistency	no serious indirectness	serious[34]	reporting bias[35]	7/16 (43.8%)	5/15 (33.3%)	OR 1.56 (0.36 to 6.69)	105 more per 1000 (from 181 fewer to 437 more)	⊕⊕OO LOW	
								33.3%		105 more per 1000 (from 181 fewer to 437 more)		
**New Outcome - RTX VS CSA (follow-up mean 24 months)**												
1	randomised trials	no serious risk of bias	no serious inconsistency	no serious indirectness	no serious imprecision[36]	reporting bias[37]	23/65 (35.4%)	0/65 (0%)	OR 72.44 (4.28 to 1224.53)	-	⊕⊕⊕O MODERATE	
								0%		-		
**New Outcome - TAC VS MMF (follow-up mean 9 months)**												
1	randomised trials	no serious risk of bias	no serious inconsistency	no serious indirectness	no serious imprecision[38]	reporting bias[39]	16/30 (53.3%)	6/30 (20%)	not pooled	not pooled	⊕⊕⊕O MODERATE	
								0%		not pooled		
**New Outcome - CSA VS CON (follow-up mean 12 months)**												
1	randomised trials	no serious risk of bias	no serious inconsistency	no serious indirectness	serious[40]	reporting bias[41]	1/10 (10%)	0/10 (0%)	OR 3.32 (0.12 to 91.6)	-	⊕⊕OO LOW	
								0%		-		
**New Outcome - CSA VS CTX (follow-up mean 9 months)**												
1	randomised trials	no serious risk of bias	no serious inconsistency	no serious indirectness	serious[42]	reporting bias[43]	1/10 (10%)	4/8 (50%)	OR 0.11 (0.01 to 1.34)	401 fewer per 1000 (from 490 fewer to 73 more)	⊕⊕OO LOW	
								50%		401 fewer per 1000 (from 490 fewer to 73 more)		
**New Outcome - RTX VS CTX (follow-up mean 24 months)**												
2	randomised trials	no serious risk of bias	serious[44]	no serious indirectness	no serious imprecision[45]	none[46]	17/80 (21.3%)	33/80 (41.3%)	OR 0.37 (0.18 to 0.75)	206 fewer per 1000 (from 68 fewer to 300 fewer)	⊕⊕⊕O MODERATE	
								39.7%		201 fewer per 1000 (from 66 fewer to 291 fewer)		

1 The study of Liang was not RCT,However the other 5 papers were all RCTs with high quality.

2 The heterogeneity of the 6 studies was high(I2=79%),more important,the results of these papers were different with each other.

3 The optimal information size(OIS) was 18986 >the toal events(417).The outcome is imprecise.(α=0.05； β=0.2； rate of treatment group=0.40； rate of control group=0.42)

4 Egger's test was used to detect the publication bias,the P value=0.002<0.1,therefore,the publication bias of these studies was significant.

5 The optimal information size(OIS) was 466 > the toal events(48).The outcome is imprecise very seriously.(α=0.05； β=0.2； rate of treatment group=0. 120； rate of control group=0.217)

6 There is only one article with samll sample in this study,so we suspect it's publication bias seriously.

7 The optimal information size(OIS) was 580 >the toal events(36).The outcome is imprecise very seriously.(α=0.05； β=0.2； rate of treatment group=0.053； rate of control group=0.118)

8 There is only one article with samll sample in this study,so we suspect it's publication bias seriously.

9 The heterogeneity of the 4 studies was low(I2=0%),all the outcomes were consistent with each other

10 The optimal information size(OIS) was 18986>the toal events(277).The outcome is imprecise seriously.(α=0.05； β=0.2； rate of treatment group=0.400； rate of control group=0.420)

11 Egger's test was used to detect the publication bias,the P value=0.92>0. 1,therefore,the publication bias of these studies was not significant

12 The optimal information size(OIS) was 262>the toal events(20).The outcome is imprecise seriously.(α=0.05； β=0.2； rate of treatment group=0.182； rate of control group=0.333)

13 There is only one article with samll sample in this study,so we suspect it's publication bias seriously

14 The optimal information size(OIS) was 8702>the toal events(39).The outcome is imprecise seriously.(α=0.05； β=0.2； rate of treatment group=0.093； rate of control group=0.209)

15 There is only one article with samll sample in this study,so we suspect it's publication bias seriously.

16 The optimal information size(OIS) was 2152>the toal events(51).The outcome is imprecise seriously.(α=0.05； β=0.2； rate of treatment group=0.071； rate of control group=0.043)

17 There is only one article with samll sample in this study,so we suspect it's publication bias seriously

18 The heterogeneity of the 2 studies was low(I2=0%),all the outcomes were consistent with each other.

19 The optimal information size(OIS) was 144>the toal events(80).The outcome is imprecise seriously.(α=0.05； β=0.2； rate of treatment group=0.158； rate of control group=0.024)

20 There are only two articles with samll sample in this study,so we suspect it’s publication bias seriously

21 he heterogeneity of the 3 studies was low(I2=23.3%),all the outcomes were consistent with each other.

22 The optimal information size(OIS) was 300 >the toal events(137).The outcome is imprecise seriously.(α=0.05； β=0.2； rate of treatment group=0.333； rate of control group=0. 191)

23 Egger’s test was used to detect the publication bias,the P value=0.644>0.1,therefore,the publication bias of these studies was not significant

24 The heterogeneity of the 2 studies was low(I2=0%),all the outcomes were consistent with each other.

25 The optimal information size(OIS) was 7060 >the toal events(230).The outcome is imprecise seriously.(α=0.05； β=0.2； rate of treatment group=0.174； rate of control group=0.200)

26 There are only two articles with samll sample in this study,so we suspect it’s publication bias seriously

27 The optimal information size(OIS) was 466 >the toal events(48).The outcome is imprecise seriously.(α=0.05； β=0.2； rate of treatment group=0. 120； rate of control group=0.217)

28 There are only two articles with samll sample in this study,so we suspect it’s publication bias seriously

29 The heterogeneity of the 3 studies was low(I2=0%),all the outcomes were consistent with each other.

30 The optimal information size(OIS) was 52 <the toal events(188).and the 95%CI excluded no effect,The outcome is precise.(α=0.05； β=0.2； rate of treatment group=0.400； rate of control group=0.075)

31 Egger’s test was used to detect the publication bias,the P value=0.548>0.1,therefore,the publication bias of these studies was not significant.

32 The optimal information size(OIS) was 112 >the toal events(75).and the 95%CI excluded no effect,The outcome is precise.(α=0.05； β=0.2； rate of treatment group=0. 189； rate of control group=0.026)

33 There is only one article with samll sample in this study,so we suspect it’s publication bias seriously.

34 The optimal information size(OIS) was 674 >the toal events(31).The outcome is imprecise seriously.(α=0.05； β=0.2； rate of treatment group=0.438； rate of control group=0.333)

35 There is only one article with samll sample in this study,so we suspect it’s publication bias seriously.

36 The optimal information size(OIS) was 5<the toal events(130).and the 95%CI excluded no effect,The outcome is precise.(α=0.05； β=0.2； rate of treatment group=0.354； rate of control group=0.00)

37 There is only one article with samll sample in this study,so we suspect it’s publication bias seriously.

38 The optimal information size(OIS) was 60=the toal events(60).The outcome is imprecise very seriously.(α=0.05； β=0.2； rate of treatment group=0.533； rate of control group=0.200)

39 There is only one article with samll sample in this study,so we suspect it’s publication bias seriously.

40 The optimal information size(OIS) was 5382>the toal events(277).The outcome is imprecise seriously.(α=0.05； β=0.2； rate of treatment group=0.254； rate of control group=0.288)

41 There are only two articles with samll sample in this study,so we suspect it’s publication bias seriously.

42 The optimal information size(OIS) was 40 >the toal events(18).The outcome is imprecise seriously.(α=0.05； β=0.2； rate of treatment group=0.10； rate of control group=0.50)

43 There is only one article with samll sample in this study,so we suspect it’s publication bias seriously.

44 The heterogeneity of the 2 studies was high (I2=65%), the outcomes were not consistent with each other.

45 The optimal information size(OIS) was 158 <the toal events(160).The outcome is imprecise seriously.(α=0.05； β=0.2； rate of treatment group=0.213； rate of control group=0.413)

46 Begg’s test was used to detect the publication bias,the P value=0.317>0.1,therefore,the publication bias of these studies was not significant.
