# Supplementary material for: Immunosuppressive treatment for idiopathic membranous nephropathy: An updated network meta-analysis
Source: Open Life Sci. 2023 Jan 10;18(1):20220527. doi: 10.1515/biol-2022-0527 (PMC9835199; doi:10.1515/biol-2022-0527)
Supplement: Supplementary Table 7 [file SupTable_7.Inconsistency_test_for_CR.pdf]

Side	Direct Coef.	Std. Err.	Indirect Coef.	Std. Err.	Difference Coef.	Std. Err.	P>z
A B	-1.55946	1.007302	-0.13077	0.655373	-1.42869	1.199264	0.234
A C	-0.44183	1.13612	-1.51601	0.829695	1.074176	1.406827	0.445
A D	-0.0648	0.389205	1.68094	0.896758	-1.74574	0.978833	0.075
A H	0.711496	1.109057	-1.73469	0.634729	2.44619	1.277845	0.056
B C	-0.16252	1.209659	-0.81309	0.874542	0.650575	1.492682	0.663
B D	0.126115	0.580151	1.99393	0.803768	-1.86782	0.989047	0.059
B F	0.81093	1.365827	0.640981	0.729831	0.169949	1.548592	0.913
B H	0.875469	1.529249	-0.8701	0.687779	1.745568	1.676795	0.298
C D	1.924103	1.395823	1.206928	0.73083	0.717175	1.569996	0.648
C E	-0.52609	1.526315	0.36292	0.97857	-0.88901	1.813074	0.624
C G	4.282693	1.636432	0.308557	0.881137	3.974136	1.858578	0.032
C H	-0.79639	1.843384	0.177824	0.812543	-0.97421	2.037054	0.632
D F	-0.82375	0.756213	0.611826	0.737616	-1.43558	1.056438	0.174
D G	-0.89821	0.657409	2.019839	1.127309	-2.91805	1.304703	0.025
D H	-1.39172	1.020746	-1.33117	0.605528	-0.06055	1.179941	0.959
E F	0.513403	0.978579	1.635164	0.834176	-1.12176	1.285872	0.383
E H	0.11906	0.728593	-0.46524	0.976891	0.584301	1.219743	0.632
F H	-2.09952	0.637121	-0.24838	0.695057	-1.85115	0.942908	0.05
G H	-2.15563	1.380726	-0.89604	0.855031	-1.25959	1.624033	0.438
A B	-1.55946	1.007302	-0.13077	0.655373	-1.42869	1.199264	0.234

**Note*：A=TAC; B=MMF; C=CSA; D=CTX; E=STE; F=CHL; G=RTX; H=CON.*

***Supplementary Table** 7 Inconsistency test for CR. If p > 0.05, there was no inconsistency among these studies, otherwise the inconsistency was present. The results of CR indicated that the p value of comparison between CSA to RTX was 0.032, and the p value of comparison between CTX to RTX was 0.025, therefore the inconsistency was significant. The inconsistency of other comparisons for CR was not significant.*
