# Supplementary material for: Immunosuppressive treatment for idiopathic membranous nephropathy: An updated network meta-analysis
Source: Open Life Sci. 2023 Jan 10;18(1):20220527. doi: 10.1515/biol-2022-0527 (PMC9835199; doi:10.1515/biol-2022-0527)
Supplement: Supplementary Table 8 [file SupTable_8.The_league_table_plot_for_CR.pdf]

Supplementary Table 8. *The league table plot for CR*

TAC	0.58 (0.19,1.72)	0.32 (0.09,1.18)	1.24 (0.60,2.57)	0.35 (0.08,1.51)	1.13 (0.35,3.72)	1.12 (0.28,4.45)	**0.32 (0.10,1.00)**
1.74 (0.58,5.20)	MMF	0.56 (0.14,2.19)	2.16 (0.82,5.65)	0.62 (0.14,2.80)	1.97 (0.57,6.84)	1.94 (0.43,8.71)	0.56 (0.16,1.92)
3.13 (0.85,11.53)	1.80 (0.46,7.10)	CSA	**3.89 (1.10,13.75)**	1.11 (0.22,5.48)	3.55 (0.80,15.65)	3.50 (0.70,17.51)	1.01 (0.24,4.21)
0.81 (0.39,1.67)	0.46 (0.18,1.21)	**0.26 (0.07,0.91)**	CTX	0.29 (0.07,1.11)	0.91 (0.32,2.58)	0.90 (0.27,3.03)	**0.26 (0.09,0.72)**
2.82 (0.66,12.00)	1.62 (0.36,7.38)	0.90 (0.18,4.45)	3.50 (0.90,13.62)	STE	3.20 (0.93,11.02)	3.15 (0.57,17.51)	0.91 (0.30,2.81)
0.88 (0.27,2.89)	0.51 (0.15,1.76)	0.28 (0.06,1.24)	1.10 (0.39,3.09)	0.31 (0.09,1.08)	CHL	0.99 (0.22,4.47)	**0.29 (0.11,0.75)**
0.90 (0.22,3.56)	0.51 (0.11,2.31)	0.29 (0.06,1.43)	1.11 (0.33,3.73)	0.32 (0.06,1.76)	1.01 (0.22,4.60)	RTX	0.29 (0.07,1.22)
**3.10 (1.00,9.57)**	1.78 (0.52,6.08)	0.99 (0.24,4.12)	**3.84 (1.39,10.62)**	1.10 (0.36,3.38)	**3.51 (1.34,9.21)**	3.46 (0.82,14.56)	CON
