# Supplementary material for: Immunosuppressive treatment for idiopathic membranous nephropathy: An updated network meta-analysis
Source: Open Life Sci. 2023 Jan 10;18(1):20220527. doi: 10.1515/biol-2022-0527 (PMC9835199; doi:10.1515/biol-2022-0527)
Supplement: Supplementary Table 9 [file SupTable_9.Details_of_GRADE_assessment_for_TR.pdf]

__Supplementary Table 9__. Details of GRADE assessment for TR

Quality assessment							No of patients		Effect		Quality	Importance
**No of studies**	**Design**	**Risk of bias**	**Inconsistency**	**Indirectness**	**Imprecision**	**Other considerations**	**PR+CR**	**Control**	**Relative (95% CI)**	**Absolute**		
**New Outcome**												
33	no methodology chosen					none	626/1022 (61.3%)	502/1002 (50.1%)	OR 1.66 (1.13 to 2.44)	124 more per 1000 (from 31 more to 209 more)		
								55.6%		119 more per 1000 (from 30 more to 197 more)		
**New Outcome - TAC vs CTX (follow-up mean 14.5 months)**												
6	randomised trials	no serious risk of bias	serious[1]	no serious indirectness	serious[2]	none[3]	149/210 (71%)	154/207 (74.4%)	OR 1.01 (0.36 to 2.82)	2 more per 1000 (from 233 fewer to 147 more)	⊕⊕OO LOW	
								74.2%		2 more per 1000 (from 233 fewer to 148 more)		
**New Outcome - TAC vs CON (follow-up mean 30 months)**												
1	randomised trials	no serious risk of bias	no serious inconsistency	no serious indirectness	serious[4]	reporting bias[5]	10/25 (40%)	7/23 (30.4%)	OR 1.52 (0.46 to 5.04)	95 more per 1000 (from 137 fewer to 384 more)	⊕⊕OO LOW	
								30.4%		95 more per 1000 (from 137 fewer to 384 more)		
**New Outcome - MMF vs CON (follow-up mean 12 months)**												
1	randomised trials	no serious risk of bias	no serious inconsistency	no serious indirectness	serious[6]	reporting bias[7]	7/19 (36.8%)	7/17 (41.2%)	OR 0.83 (0.22 to 3.19)	44 fewer per 1000 (from 278 fewer to 279 more)	⊕⊕OO LOW	
								41.2%		44 fewer per 1000 (from 278 fewer to 279 more)		
**New Outcome - MMF vs CTX (follow-up mean 15.75 months)**												
4	randomised trials	no serious risk of bias	serious[8]	no serious indirectness	serious[9]	none[10]	47/71 (66.2%)	52/66 (78.8%)	OR 0.59 (0.19 to 1.83)	101 fewer per 1000 (from 374 fewer to 84 more)	⊕⊕OO LOW	
								76.7%		107 fewer per 1000 (from 382 fewer to 91 more)		
**New Outcome - MMF vs CH (follow-up mean 15 months)**												
1	randomised trials	no serious risk of bias	no serious inconsistency	no serious indirectness	serious[11]	reporting bias[12]	5/11 (45.5%)	5/9 (55.6%)	OR 0.67 (0.11 to 3.92)	100 fewer per 1000 (from 435 fewer to 275 more)	⊕⊕OO LOW	
								55.6%		100 fewer per 1000 (from 435 fewer to		

										275 more)		
**New Outcome - MMF vs CSA (follow-up mean 11.2 months)**												
1	randomised trials	no serious risk of bias	no serious inconsistency	no serious indirectness	serious[13]	reporting bias[14]	16/21 (76.2%)	12/18 (66.7%)	OR 1.6 (0.39 to 6.51)	95 more per 1000 (from 228 fewer to 262 more)	⊕⊕OO LOW	
								66.7%		95 more per 1000 (from 228 fewer to 262 more)		
**New Outcome - CSA vs STE (follow-up mean 18 months)**												
1	randomised trials	no serious risk of bias	no serious inconsistency	no serious indirectness	serious[15]	reporting bias[16]	11/28 (39.3%)	3/23 (13%)	OR 4.31 (1.03 to 18.04)	262 more per 1000 (from 3 more to 600 more)	⊕⊕OO LOW	
								13%		262 more per 1000 (from 3 more to 599 more)		
**New Outcome - CTX vs CON (follow-up mean 52 months)**												
3	randomised trials	no serious risk of bias	no serious inconsistency[17]	no serious indirectness	no serious imprecision[18]	none[19]	54/85 (63.5%)	32/88 (36.4%)	OR 4.06 (2.01 to 8.19)	335 more per 1000 (from 171 more to 460 more)	⊕⊕⊕⊕ HIGH	
								26.9%		330 more per 1000 (from 156 more to 482 more)		
**New Outcome - CTX vs CH (follow-up mean 22.75 months)**												
3	randomised trials	no serious risk of bias	serious[20]	no serious indirectness	serious[21]	none[22]	54/69 (78 .3%)	42/68 (61 .8%)	OR 2.89 (0.63 to 13.27)	206 more per 1000 (from 113 fewer to 338 more)	⊕⊕OO LOW	
								44.4%		254 more per 1000 (from 109 fewer to 470 more)		
**New Outcome - STE vs CON (follow-up mean 35.5 months)**												
2	randomised trials	no serious risk of bias	no serious inconsistency[23]	no serious indirectness	serious[24]	none[25]	19/86 (22.1%)	11/89 (12.4%)	OR 2.15 (0.94 to 4.94)	109 more per 1000 (from 7 fewer to 287 more)	⊕⊕⊕O MODERATE	
								13.1%		114 more per 1000 (from 7 fewer to 296 more)		
**New Outcome - CH vs STE (follow-up mean 48 months)**												
1	randomised trials	no serious risk of bias	no serious inconsistency	no serious indirectness	serious[26]	reporting bias[27]	28/45 (62.2%)	18/47 (38.3%)	OR 2.65 (1.14 to 6.16)	239 more per 1000 (from 31 more to 410 more)	⊕⊕OO LOW	
								38.3%		239 more per 1000 (from 31 more to 410 more)		
**New Outcome - CH vs CON (follow-up mean 49.6 months)**												
3	randomised trials	no serious risk of bias	no serious inconsistency[28]	no serious indirectness	no serious imprecision[29]	none[30]	66/95 (69.5%)	31/93 (33.3%)	OR 4.65 (2.49 to 8.68)	366 more per 1000 (from 221 more to 479 more)	⊕⊕⊕⊕ HIGH	
										366 more		

								33.3%		per 1000 (from 221 more to 480 more)		
**New Outcome - RTX vs CON (follow-up mean 17 months)**												
1	randomised trials	no serious risk of bias	no serious inconsistency	no serious indirectness	serious[31]	reporting bias[32]	24/37 (64.9%)	13/38 (34.2%)	OR 3.55 (1.37 to 9.19)	307 more per 1000 (from 74 more to 485 more)	⊕⊕OO LOW	
								34.2%		307 more per 1000 (from 74 more to 485 more)		
**New Outcome - TAC vs CSA (follow-up mean 6 months)**												
1	randomised trials	no serious risk of bias	no serious inconsistency	no serious indirectness	serious[33]	reporting bias[34]	14/16 (87.5%)	11/15 (73.3%)	OR 2.55 (0.39 to 16.55)	142 more per 1000 (from 216 fewer to 245 more)	⊕⊕OO LOW	
								73.3%		142 more per 1000 (from 216 fewer to 245 more)		
**New Outcome - RTX vs CSA (follow-up mean 24 months)**												
1	randomised trials	no serious risk of bias	no serious inconsistency	no serious indirectness	no serious imprecision[35]	reporting bias[36]	39/65 (60%)	13/65 (20%)	OR 6 (2.74 to 13.15)	400 more per 1000 (from 207 more to 567 more)	⊕⊕⊕O MODERATE	
								20%		400 more per 1000 (from 207 more to 567 more)		
**New Outcome - TAC vs MMF (follow-up mean 9 months)**												
1	randomised trials	no serious risk of bias	no serious inconsistency	no serious indirectness	serious[37]	reporting bias[38]	25/30 (83.3%)	21/30 (70%)	OR 2.14 (0.62 to 7.39)	133 more per 1000 (from 109 fewer to 245 more)	⊕⊕OO LOW	
								70%		133 more per 1000 (from 109 fewer to 245 more)		
**New Outcome - CSA vs CON (follow-up mean 12 months)**												
1	randomised trials	no serious risk of bias	serious	no serious indirectness	serious[39]	reporting bias[40]	10/19 (52.6%)	10/18 (55.6%)	OR 0.97 (0.01 to 177.01)	8 fewer per 1000 (from 543 fewer to 440 more)	⊕OOO VERY LOW	
								50%		8 fewer per 1000 (from 490 fewer to 494 more)		
**New Outcome - CSA vs CTX (follow-up mean 9 months)**												
1	randomised trials	no serious risk of bias	no serious inconsistency	no serious indirectness	serious[41]	reporting bias[42]	6/10 (60%)	8/8 (100%)	OR 0.08 (0 to 1.88)	-	⊕⊕OO LOW	
								100%		-		
**New Outcome - RTX vs CTX (follow-up mean 24 months)**												
2	randomised trials	no serious risk of bias	no serious inconsistency[43]	no serious indirectness	serious[44]	none[45]	42/80 (52.5%)	52/80 (65%)	OR 0.56 (0.14 to 2.24)	140 fewer per 1000 (from 444 fewer to 156 more)	⊕⊕⊕O MODERATE	
								63.5%		142 fewer per 1000 (from 439 fewer to		

										161 more)		

[1] The heterogeneity of the 6 studies was high(I2=79%),more important,the results of these papers were different with each other.

[2] The optimal information size(OIS) was 5388 >the toal events(417).The outcome is imprecise.(α=0.05； β=0.2； rate of treatment group=0.710； rate of control group=0.744)

[3] Egger's test was used to detect the publication bias,the P value=0.13>0.1,therefore,the publication bias of these studies was not significant

[4] The optimal information size(OIS) was 776 > the toal events(48).The outcome is imprecise very seriously.(α=0.05； β=0.2； rate of treatment group=0.400； rate of control group=0.304)

[5] There is only one article with samll sample in this study,so we suspect it's publication bias seriously

[6] The optimal information size(OIS) was 3856 > the toal events(36).The outcome is imprecise very seriously.(α=0.05； β=0.2； rate of treatment group=0.368； rate of control group=0.412)

[7] There is only one article with samll sample in this study,so we suspect it's publication bias seriously

[8] The heterogeneity of the 4 studies was high(I2=37.4%),more important,the results of these papers were different with each other

[9] The optimal information size(OIS) was 392 > the toal events(137).The outcome is imprecise very seriously.(α=0.05； β=0.2； rate of treatment group=0.662； rate of control group=0.788)

[10] Egger's test was used to detect the publication bias,the P value=0.265>0.1,therefore,the publication bias of these studies was not significant

[11] The optimal information size(OIS) was 768>the toal events(20).The outcome is imprecise seriously.(α=0.05； β=0.2； rate of treatment group=0.455； rate of control group=0.556)

[12] There is only one article with samll sample in this study,so we suspect it's publication bias seriously

[13] The optimal information size(OIS) was 708>the toal events(39).The outcome is imprecise seriously.(α=0.05； β=0.2； rate of treatment group=0.762； rate of control group=0.667)

[14] There is only one article with samll sample in this study,so we suspect it's publication bias seriously

[15] The optimal information size(OIS) was 86 >the toal events(51).The outcome is imprecise very seriously.(α=0.05； β=0.2； rate of treatment group=0.393； rate of control group=0.130)

[16] There is only one article with samll sample in this study,so we suspect it's publication bias seriously

[17] The heterogeneity of the 3 studies was low(I2=0%),all the outcomes were consistent with each other

[18] The optimal information size(OIS) was 106 <the toal events(173).and the 95%CI excluded no effect,The outcome is precise.(α=0.05； β=0.2； rate of treatment group=0.635； rate of control group=0.364)

[19] Egger's test was used to detect the publication bias,the P value=0.187>0.1,therefore,the publication bias of these studies was not significant

[20] The heterogeneity of the 3 studies was high(I2=58.9%),more important,the results of these papers were different with each other.

[21] The optimal information size(OIS) was 240>the toal events(137).The outcome is imprecise seriously.(α=0.05； β=0.2； rate of treatment group=0.783； rate of control group=0.618)

[22] Egger's test was used to detect the publication bias,the P value=0.880>0.1,therefore,the publication bias of these studies was not significant

[23] The heterogeneity of the 2 studies was low(I2=0%),all the outcomes were consistent with each other

[24] The optimal information size(OIS) was 474>the toal events(175).The outcome is imprecise seriously.(α=0.05； β=0.2； rate of treatment group=0.221； rate of control group=0.124)

[25] Begg's test was used to detect the publication bias,the P value=0.317>0. 1,therefore,the publication bias of these studies was not significant

[26] The optimal information size(OIS) was 132>the toal events(92).The outcome is imprecise seriously.(α=0.05； β=0.2； rate of treatment group=0.622； rate of control group=0.383)

[27] There is only one article with samll sample in this study,so we suspect it's publication bias seriously

[28] The heterogeneity of the 4 studies was low(I2=0%),all the outcomes were consistent with each other

[29] The optimal information size(OIS) was 58 <the toal events(188).and the 95%CI excluded no effect,The outcome is precise.(α=0.05； β=0.2； rate of treatment group=0.695； rate of control group=0.333)

[30] Egger's test was used to detect the publication bias,the P value=0.257>0.1,therefore,the publication bias of these studies was not significant.

[31] The optimal information size(OIS) was 82 >the toal events(75).and the 95%CI excluded no effect,The outcome is precise.(α=0.05； β=0.2； rate of treatment group=0.645； rate of control group=0.349)

[32] There is only one article with samll sample in this study,so we suspect it's publication bias seriously

[33] The optimal information size(OIS) was 244 >the toal events(31).The outcome is imprecise seriously.(α=0.05； β=0.2； rate of treatment group=0.875； rate of control group=0.733)

[34] There is only one article with samll sample in this study,so we suspect it's publication bias seriously

[35] The optimal information size(OIS) was 46<the toal events(130).and the 95%CI excluded no effect,The outcome is precise.(α=0.05； β=0.2； rate of treatment group=0.600； rate of control group=0.200)

[36] There is only one article with samll sample in this study,so we suspect it's publication bias seriously

[37] The optimal information size(OIS) was 316>the toal events(60).The outcome is imprecise seriously.(α=0.05； β=0.2； rate of treatment group=0.833； rate of control group=0.700)

[38] There is only one article with samll sample in this study,so we suspect it's publication bias seriously

[39] The optimal information size(OIS) was 4862>the toal events(37).The outcome is imprecise seriously.(α=0.05； β=0.2； rate of treatment group=0.526； rate of control group=0.556)

[40] There are only two articles with samll sample in this study,so we suspect it's publication bias seriously

[41] The optimal information size(OIS) was 30 >the toal events(18).The outcome is imprecise seriously.(α=0.05； β=0.2； rate of treatment group=0.600； rate of control group=1.000)

[42] There is only one article with samll sample in this study,so we suspect it's publication bias seriously

[43] The heterogeneity of the 3 studies was low(I2=0%),all the outcomes were consistent with each other.

[44] The optimal information size(OIS) was 486 >the toal events(160).The outcome is imprecise seriously.(α=0.05； β=0.2； rate of treatment group=0.525； rate of control group=0.650)

[45] Begg's test was used to detect the publication bias,the P value=0.317>0. 1,therefore,the publication bias of these studies was not significant.
