# Supplementary material for: Immunosuppressive treatment for idiopathic membranous nephropathy: An updated network meta-analysis
Source: Open Life Sci. 2023 Jan 10;18(1):20220527. doi: 10.1515/biol-2022-0527 (PMC9835199; doi:10.1515/biol-2022-0527)
Supplement: Supplementary Table 10 [file SupTable_10.Inconsistency_test_for_TR.pdf]

| Side | Direct Coef. | Std. Err. | Indirect Coef. | Std. Err. | Difference Coef. | Std. Err. | P>z |
|---|---|---|---|---|---|---|---|
| A B | -0.71619 | 0.974732 | -0.64188 | 0.615518 | -0.07432 | 1.155001 | 0.949 |
| A C | -0.93431 | 1.206271 | -1.11561 | 0.653787 | 0.181302 | 1.372052 | 0.895 |
| A D | 0.037191 | 0.379335 | 1.085854 | 0.782493 | -1.04866 | 0.866706 | 0.226 |
| A H | -0.42121 | 0.939407 | -1.56943 | 0.505817 | 1.148215 | 1.066928 | 0.282 |
| B C | -0.47 | 1.031289 | -0.38361 | 0.716929 | -0.08639 | 1.256003 | 0.945 |
| B D | 0.525737 | 0.578023 | 1.364528 | 0.657466 | -0.83879 | 0.878146 | 0.339 |
| B F | 0.405465 | 1.166447 | 0.505904 | 0.603426 | -0.10044 | 1.313286 | 0.939 |
| B H | 0.182322 | 0.988354 | -0.91283 | 0.550634 | 1.095151 | 1.131389 | 0.333 |
| C D | 3.14454 | 1.664835 | 1.091932 | 0.554142 | 2.052608 | 1.767209 | 0.245 |
| C E | -1.4618 | 0.977797 | 0.749022 | 0.741753 | -2.21082 | 1.227308 | 0.072 |
| C G | 1.791759 | 0.80339 | 0.483121 | 0.752543 | 1.308638 | 1.100798 | 0.235 |
| C H | 0.113747 | 1.206067 | -0.32715 | 0.597965 | 0.440896 | 1.355902 | 0.745 |
| D F | -1.07421 | 0.636804 | 0.095731 | 0.550243 | -1.16994 | 0.841513 | 0.164 |
| D G | -0.57791 | 0.617749 | 0.280558 | 0.723903 | -0.85847 | 0.951574 | 0.367 |
| D H | -1.14134 | 0.568653 | -1.82889 | 0.47735 | 0.687545 | 0.744048 | 0.355 |
| E F | 0.975915 | 0.865011 | 0.968736 | 0.717996 | 0.007179 | 1.124171 | 0.995 |
| E H | -0.75318 | 0.656088 | 0.572125 | 0.7397 | -1.32531 | 0.988555 | 0.18 |
| F H | -1.60096 | 0.526076 | -0.58818 | 0.5756 | -1.01278 | 0.780368 | 0.194 |
| G H | -1.26703 | 0.89372 | -1.35663 | 0.63393 | 0.089596 | 1.09572 | 0.935 |

**Note：A=TAC; B=MMF; C=CSA; D=CTX; E=STE; F=CHL; G=RTX; H=CON.*

***Supplementary Table 10*** *Inconsistency test for TR. If p > 0.05, there was no inconsistency among these studies, otherwise the inconsistency was present. The results indicated that there was no inconsistency in all comparisons between these groups.*
