# Supplementary material for: Immunosuppressive treatment for idiopathic membranous nephropathy: An updated network meta-analysis
Source: Open Life Sci. 2023 Jan 10;18(1):20220527. doi: 10.1515/biol-2022-0527 (PMC9835199; doi:10.1515/biol-2022-0527)
Supplement: Supplementary Table 11 [file SupTable_11.The_league_table_plot_for_TR.pdf]

Supplementary Table 11. *The league table plot for TR*

**TAC**	0.52<br>(0.19,1.41)	0.34<br>(0.11,1.04)	1.26<br>(0.64,2.48)	0.32<br>(0.09,1.08)	0.84<br>(0.31,2.30)	1.02<br>(0.34,3.02)	**0.27**<br>**(0.11,0.65)**
1.93<br>(0.71,5.24)	**MMF**	0.66<br>(0.21,2.05)	**2.44**<br>**(1.03,5.74)**	0.61<br>(0.18,2.15)	1.62<br>(0.58,4.57)	1.96<br>(0.61,6.29)	0.52<br>(0.20,1.34)
2.93<br>(0.96,8.89)	1.52<br>(0.49,4.71)	**CSA**	**3.70**<br>**(1.33,10.24)**	0.93<br>(0.28,3.15)	2.46<br>(0.77,7.85)	**2.98**<br>**(1.00,8.91)**	0.79<br>(0.28,2.21)
0.79<br>(0.40,1.56)	**0.41**<br>**(0.17,0.97)**	**0.27**<br>**(0.10,0.75)**	**CTX**	**0.25**<br>**(0.08,0.76)**	0.67<br>(0.29,1.53)	0.81<br>(0.32,2.01)	**0.21**<br>**(0.11,0.43)**
3.14<br>(0.93,10.63)	1.63<br>(0.46,5.70)	1.07<br>(0.32,3.62)	**3.97**<br>**(1.32,11.92)**	**STE**	2.64<br>(0.92,7.56)	3.20<br>(0.88,11.56)	0.85<br>(0.32,2.24)
1.19<br>(0.43,3.26)	0.62<br>(0.22,1.73)	0.41<br>(0.13,1.29)	1.50<br>(0.65,3.44)	0.38<br>(0.13,1.08)	**CHL**	1.21<br>(0.39,3.74)	**0.32**<br>**(0.15,0.69)**
0.98<br>(0.33,2.91)	0.51<br>(0.16,1.63)	**0.34**<br>**(0.11,1.00)**	1.24<br>(0.50,3.10)	0.31<br>(0.09,1.13)	0.83<br>(0.27,2.56)	**RTX**	**0.26**<br>**(0.10,0.71)**
**3.71**<br>**(1.55,8.90)**	1.92<br>(0.75,4.94)	1.27<br>(0.45,3.56)	**4.69**<br>**(2.32,9.47)**	1.18<br>(0.45,3.12)	**3.12**<br>**(1.45,6.71)**	**3.78**<br>**(1.41,10.09)**	**CON**
